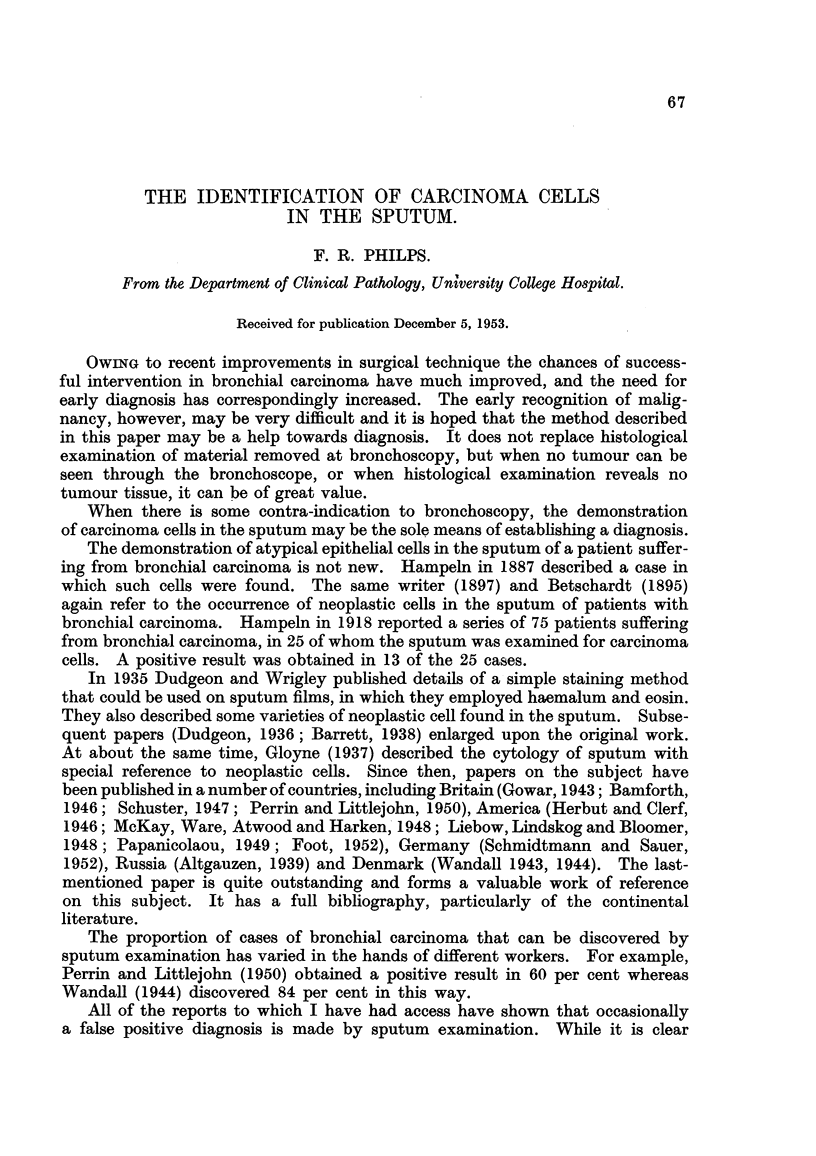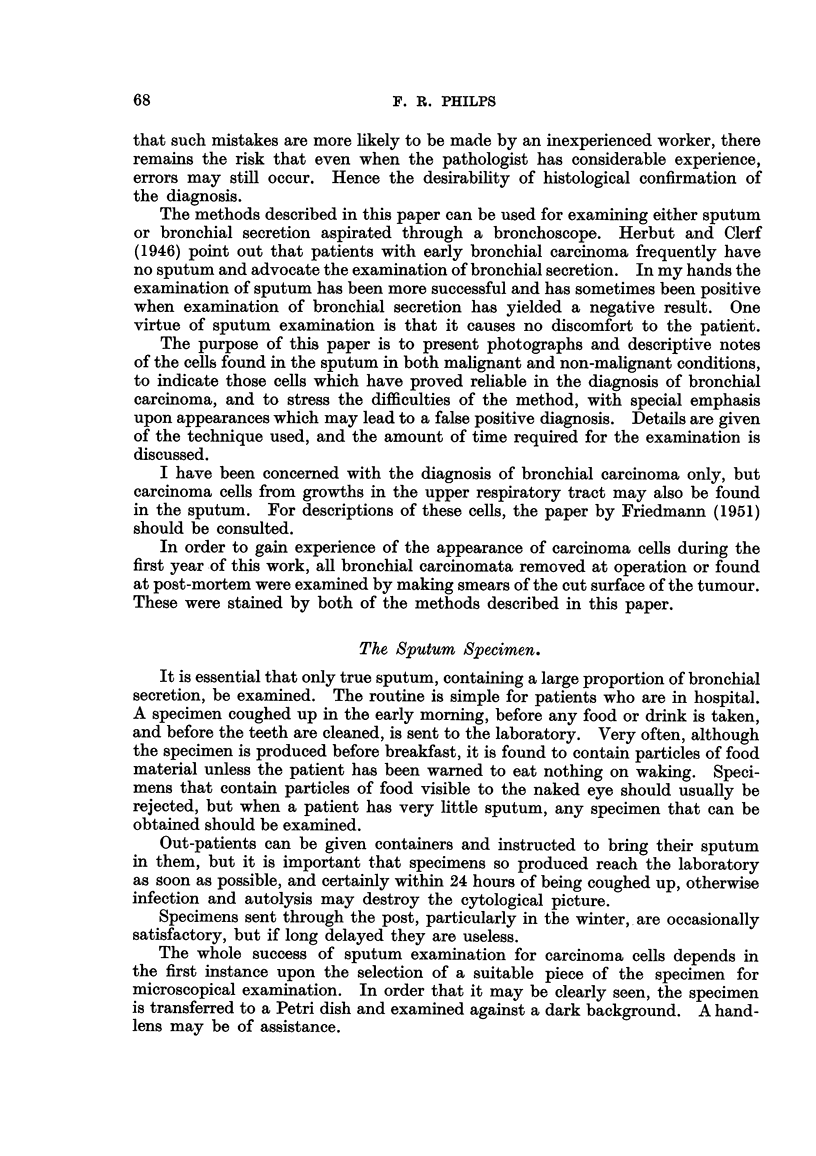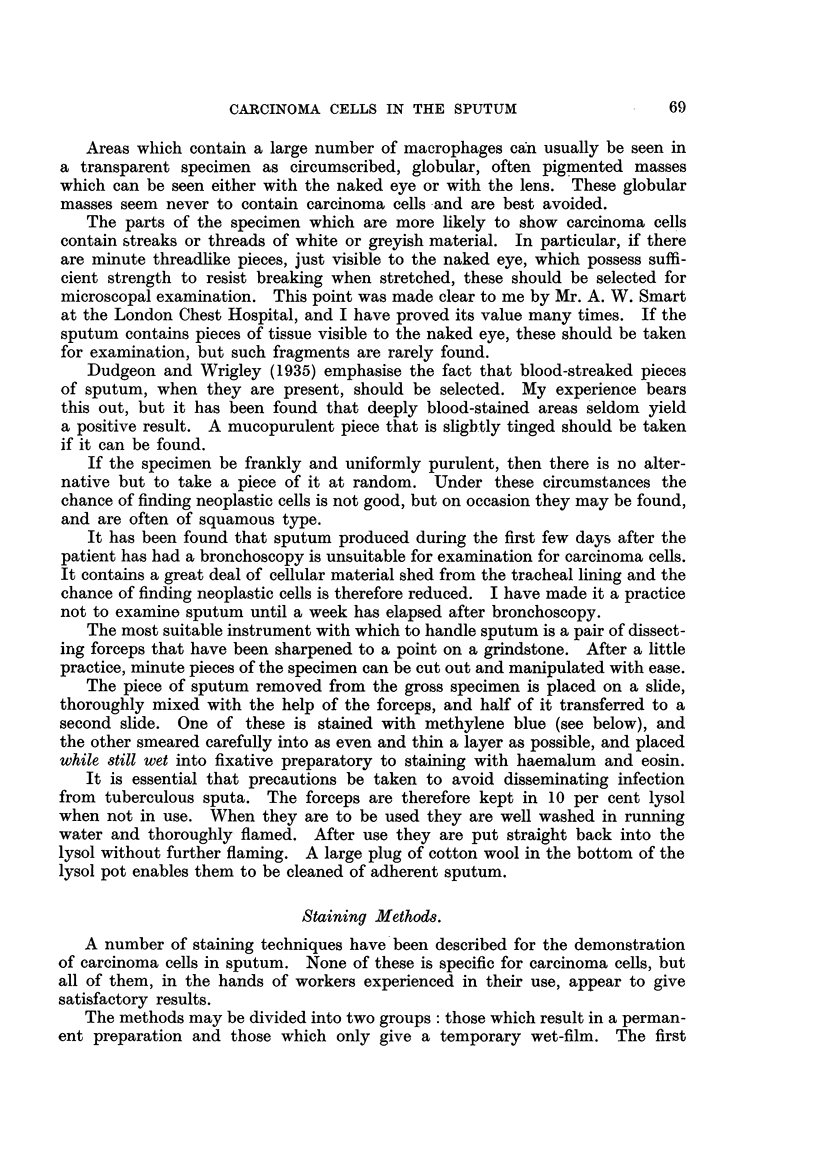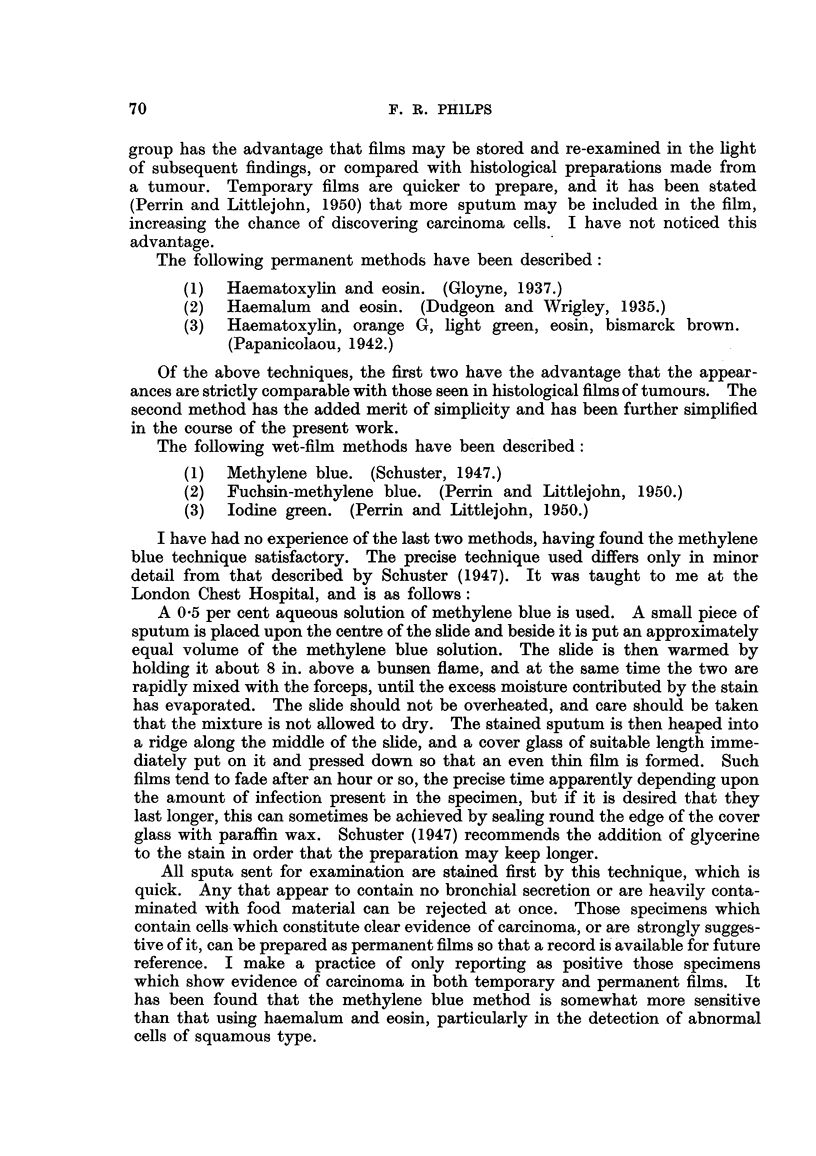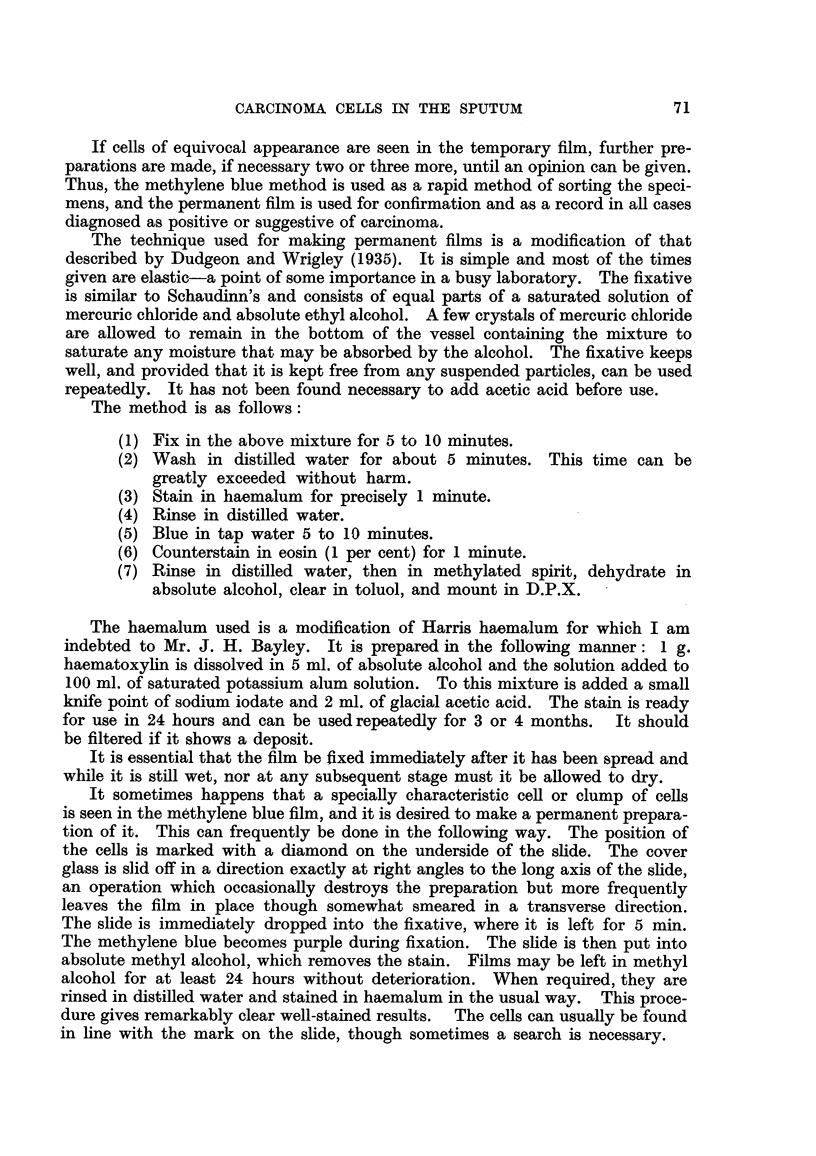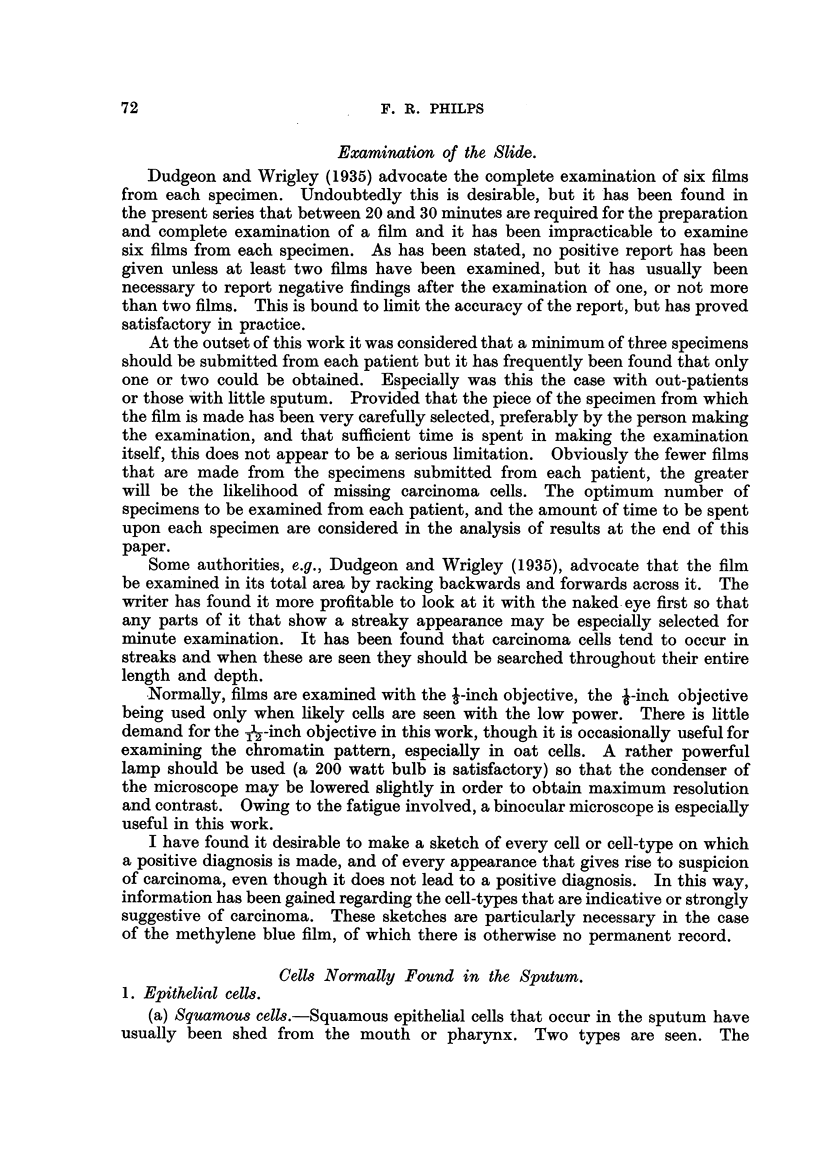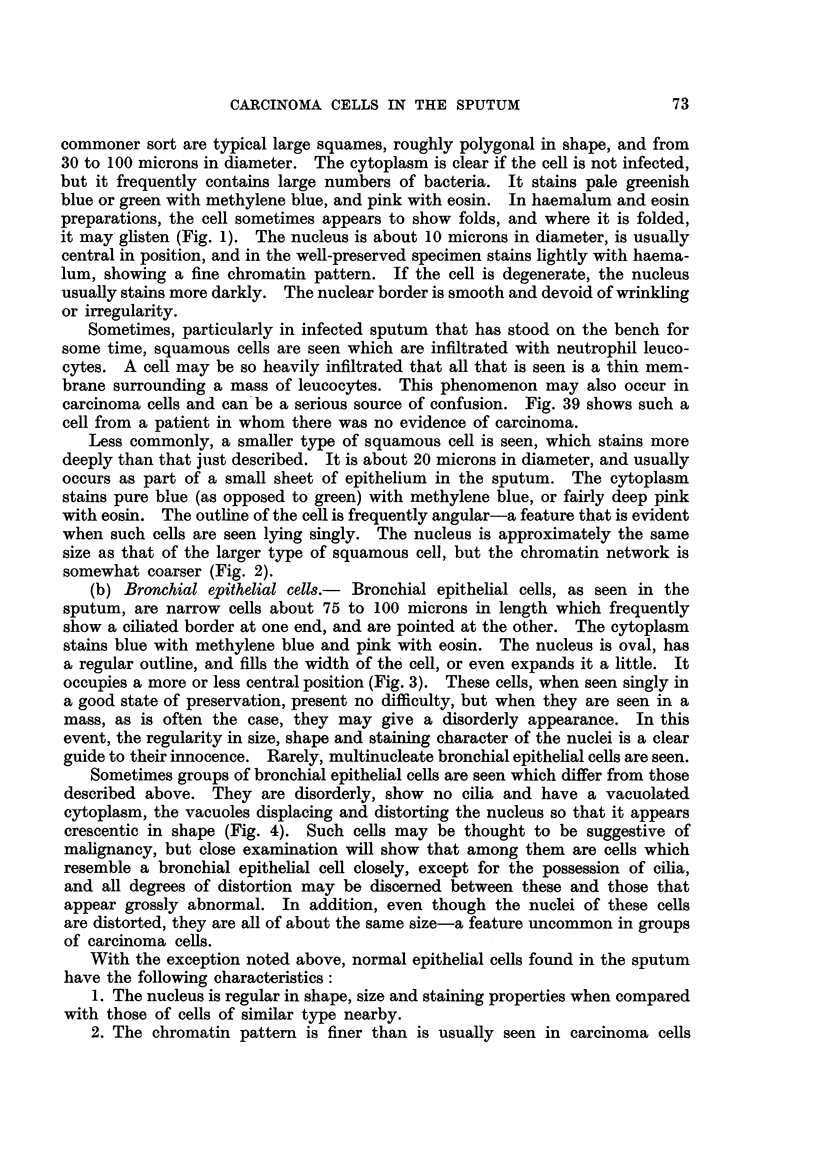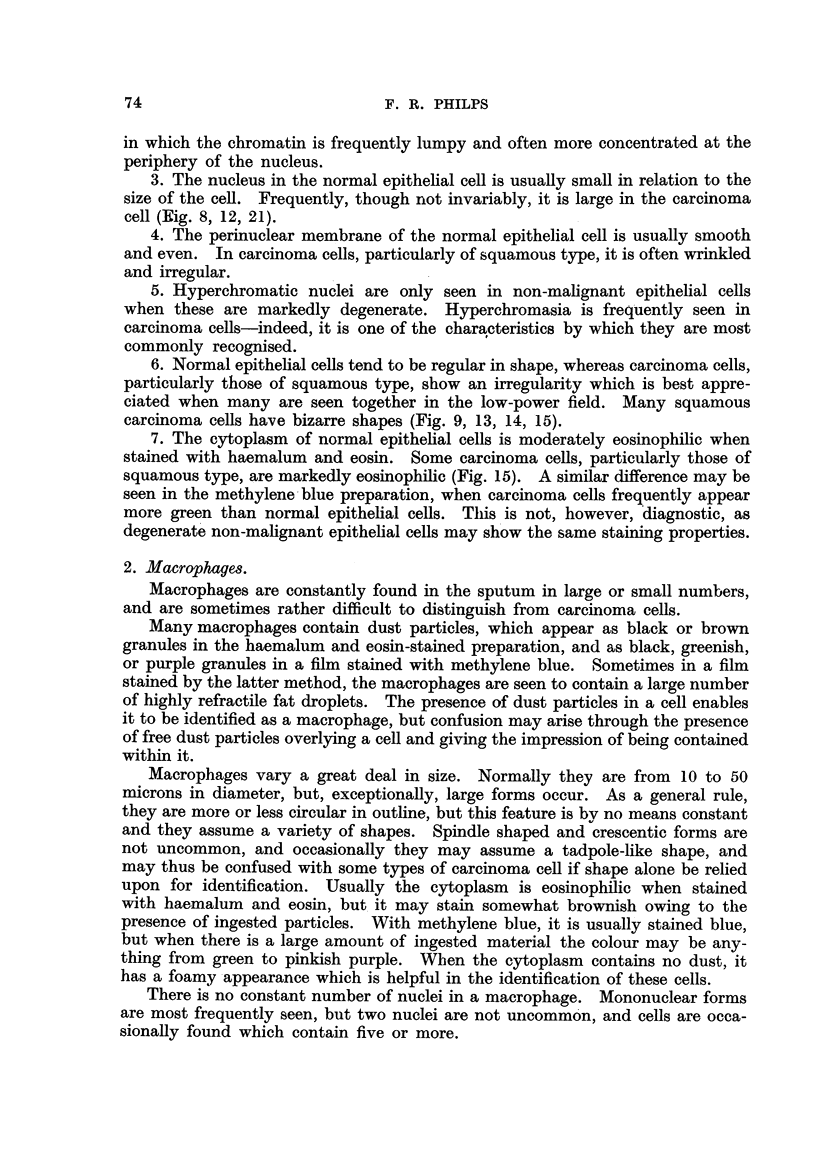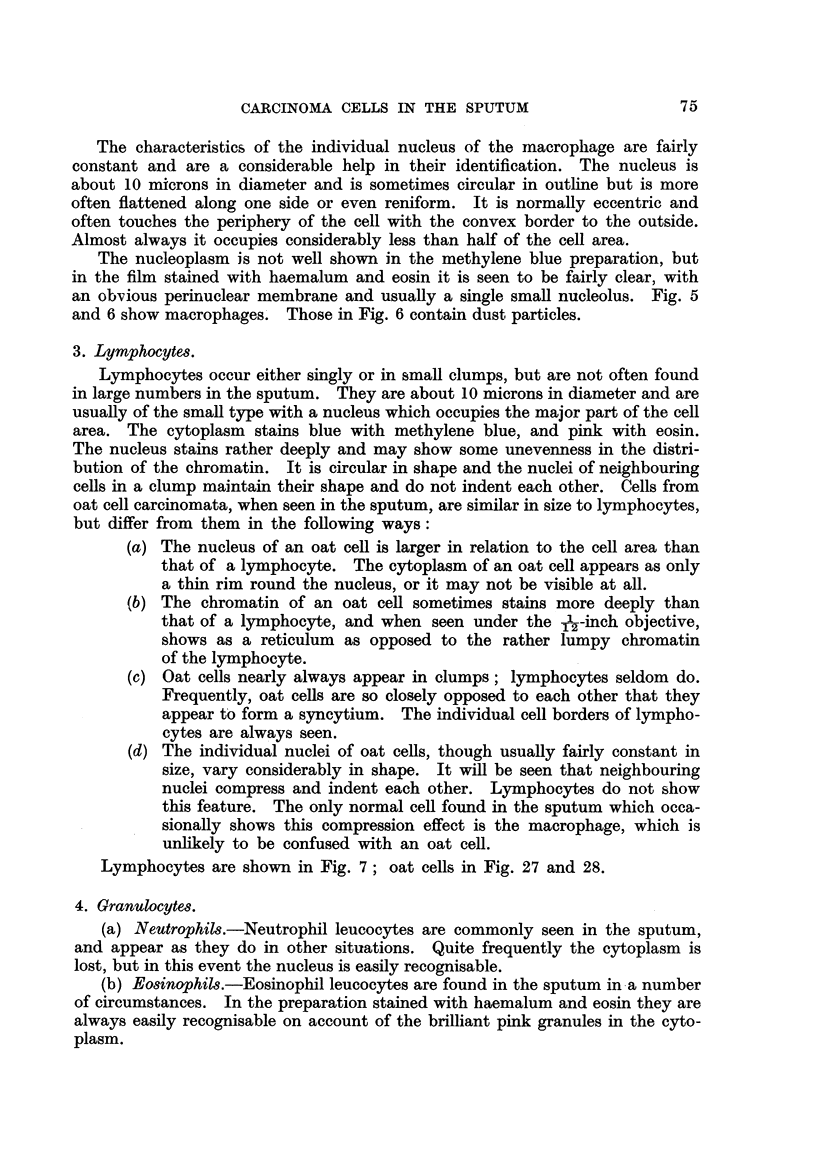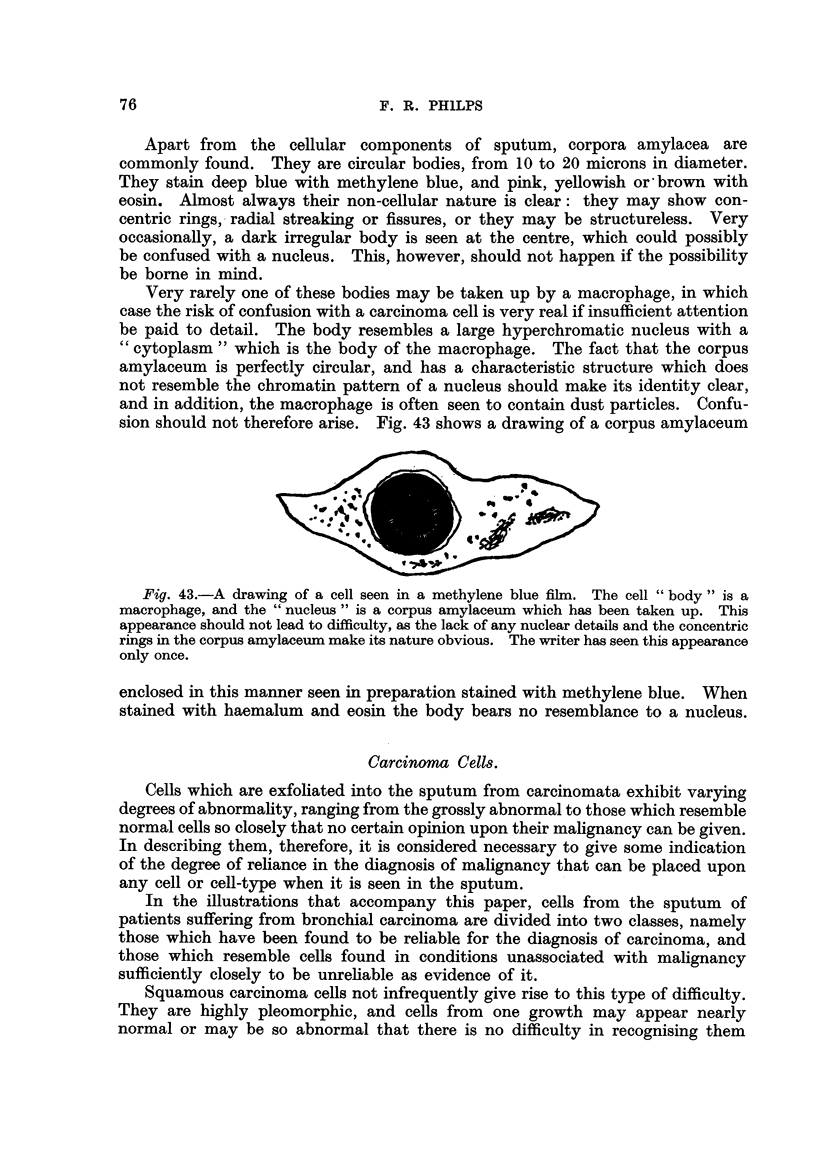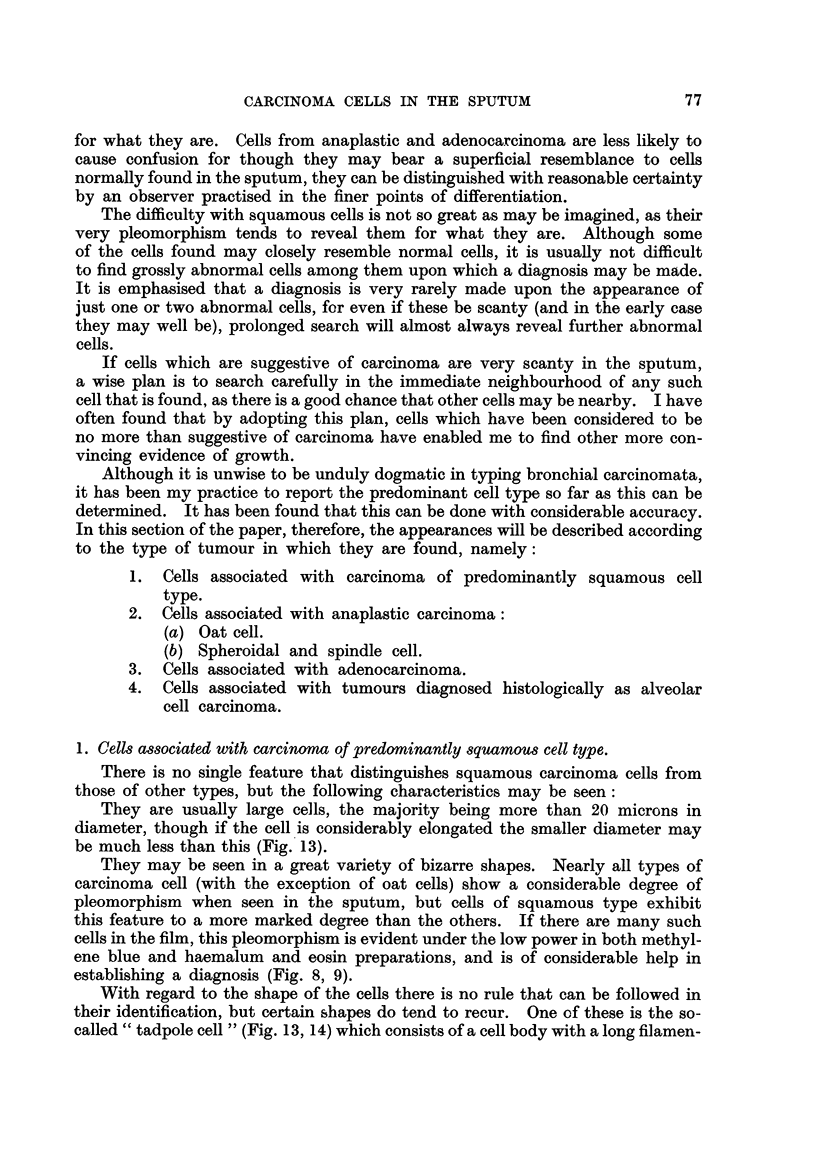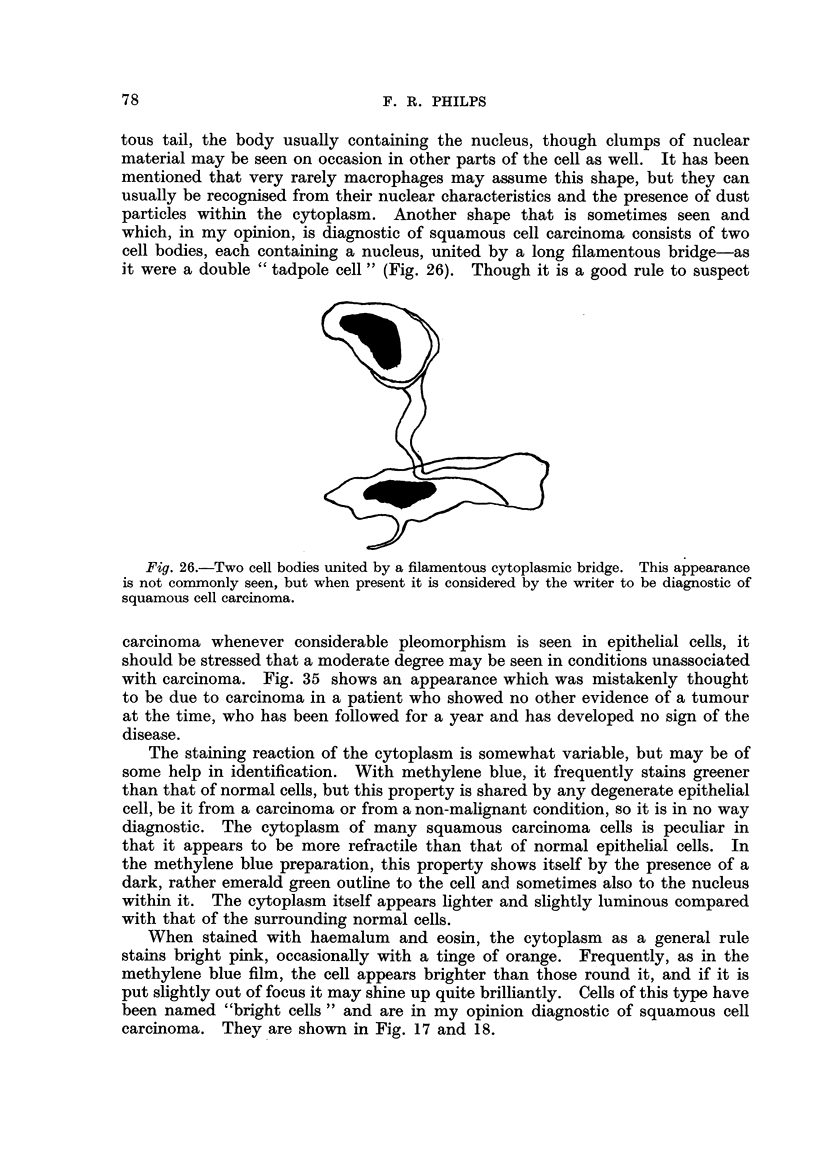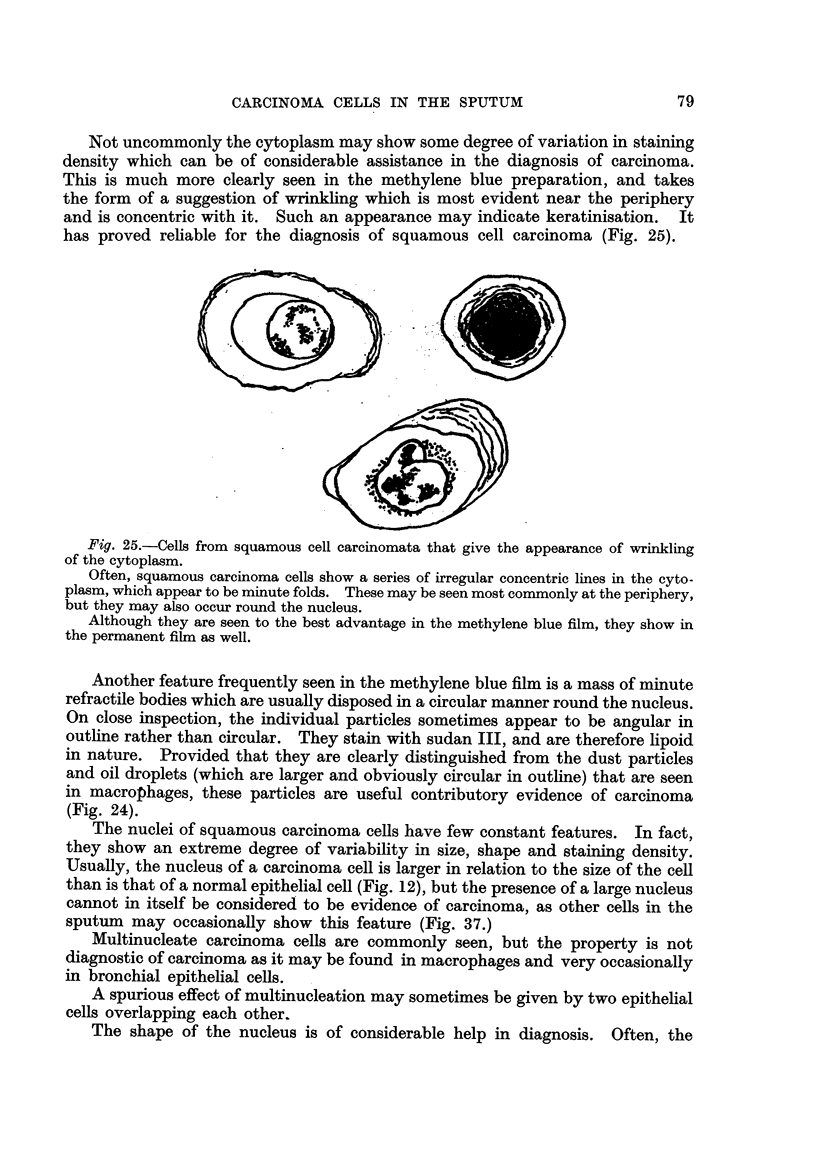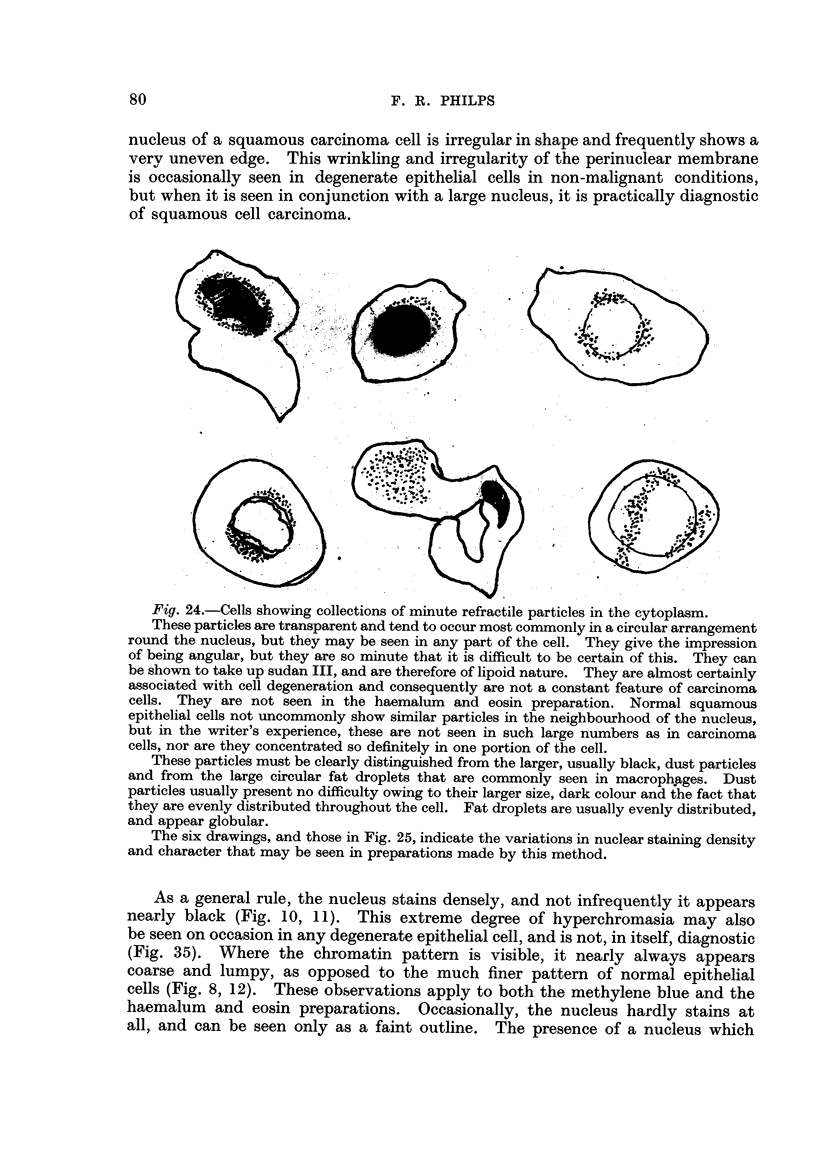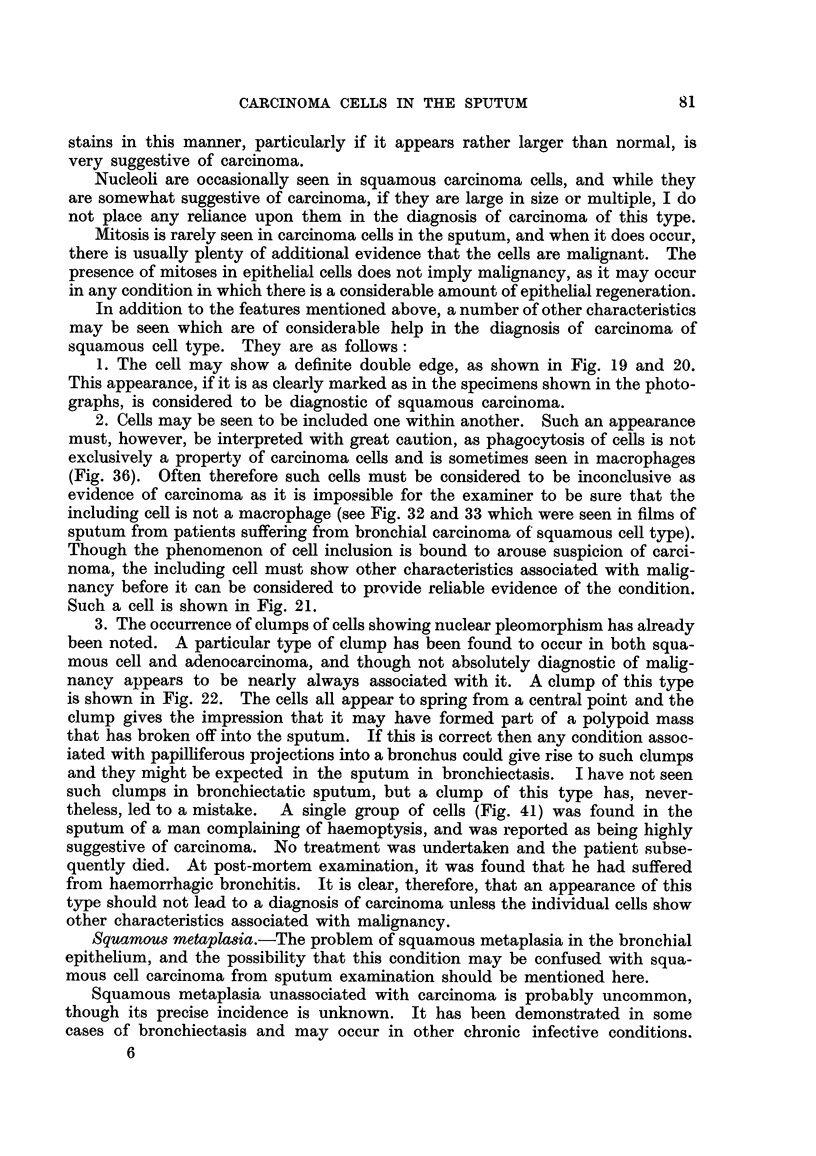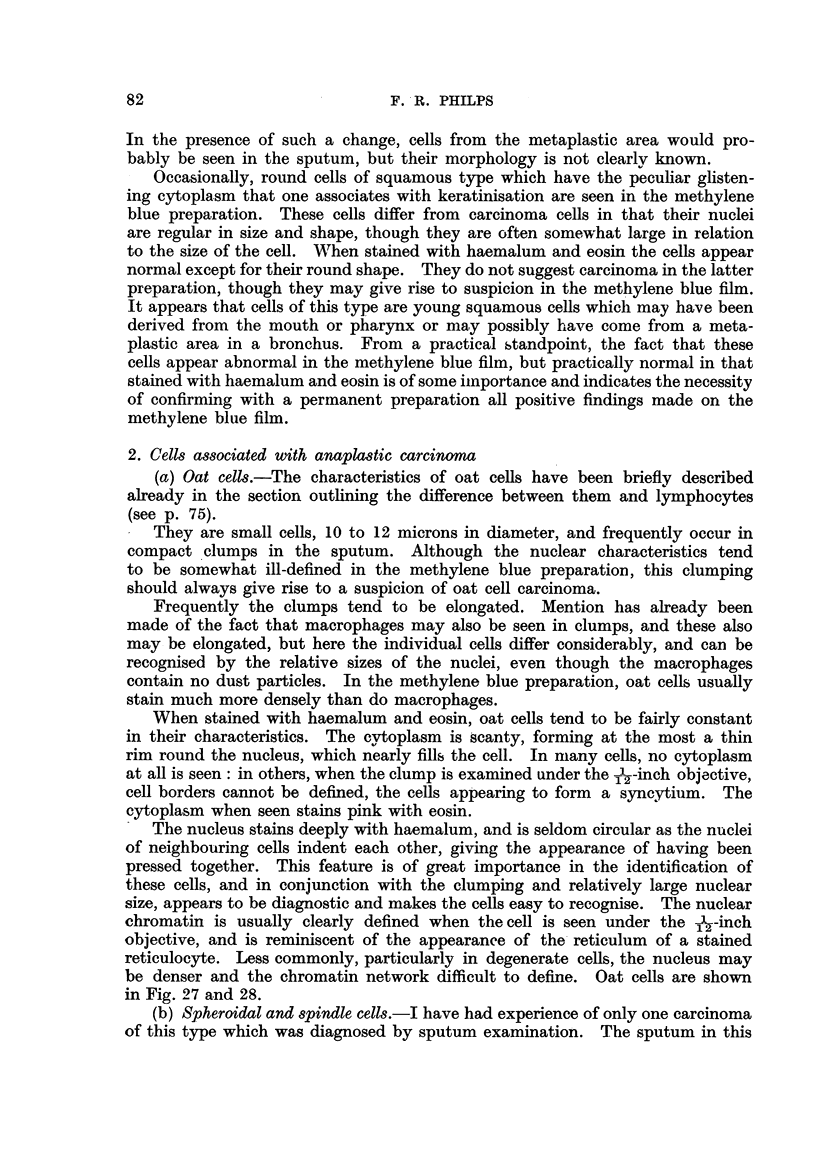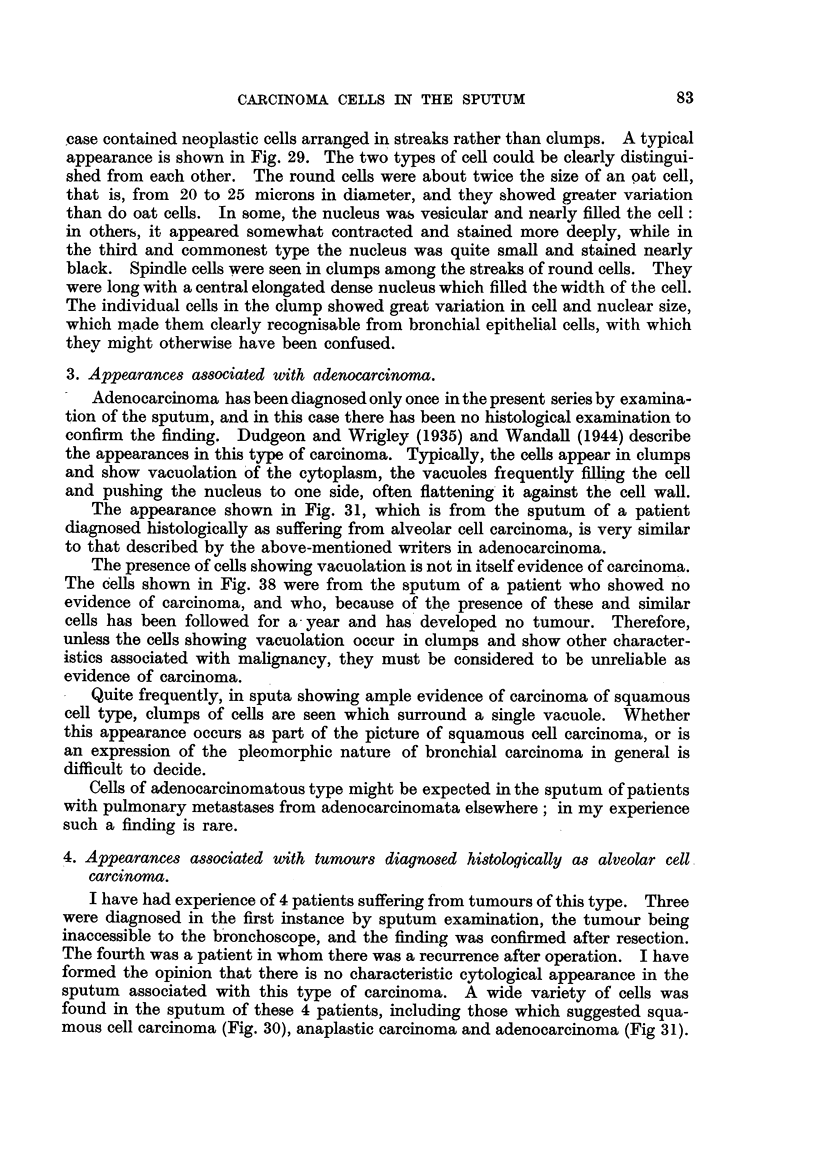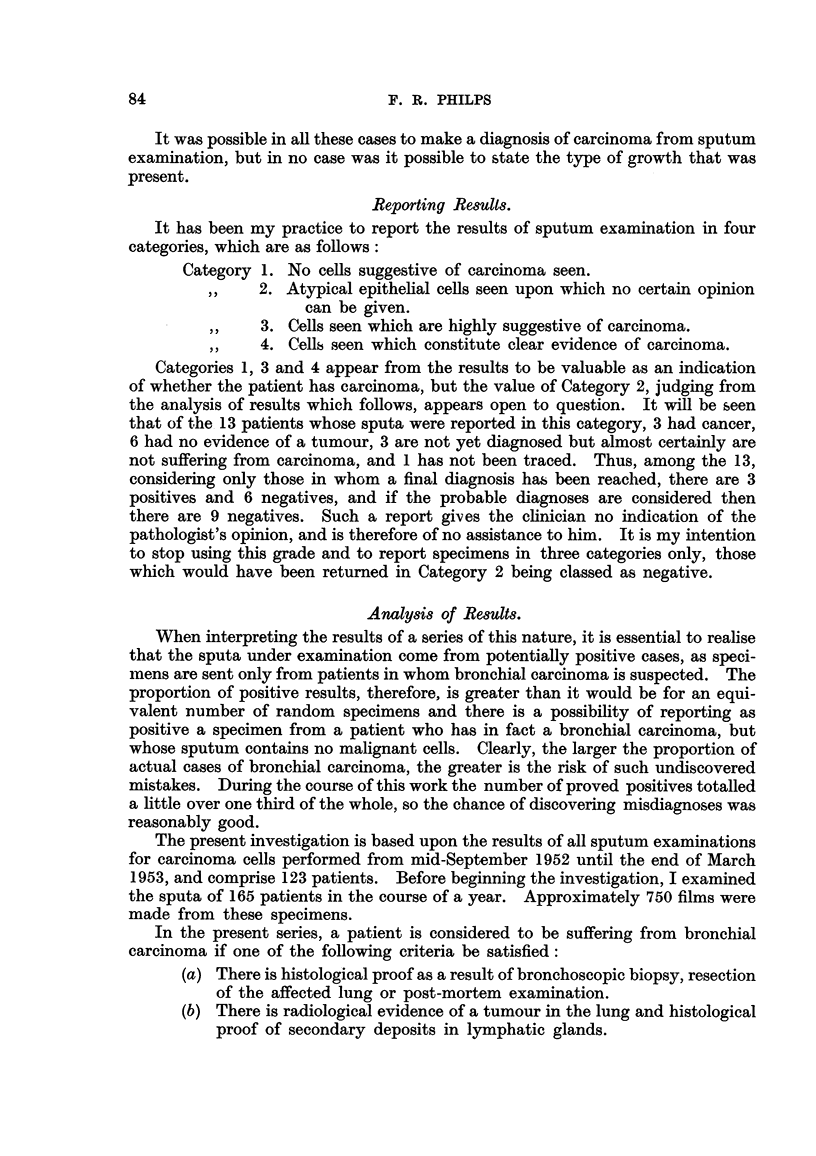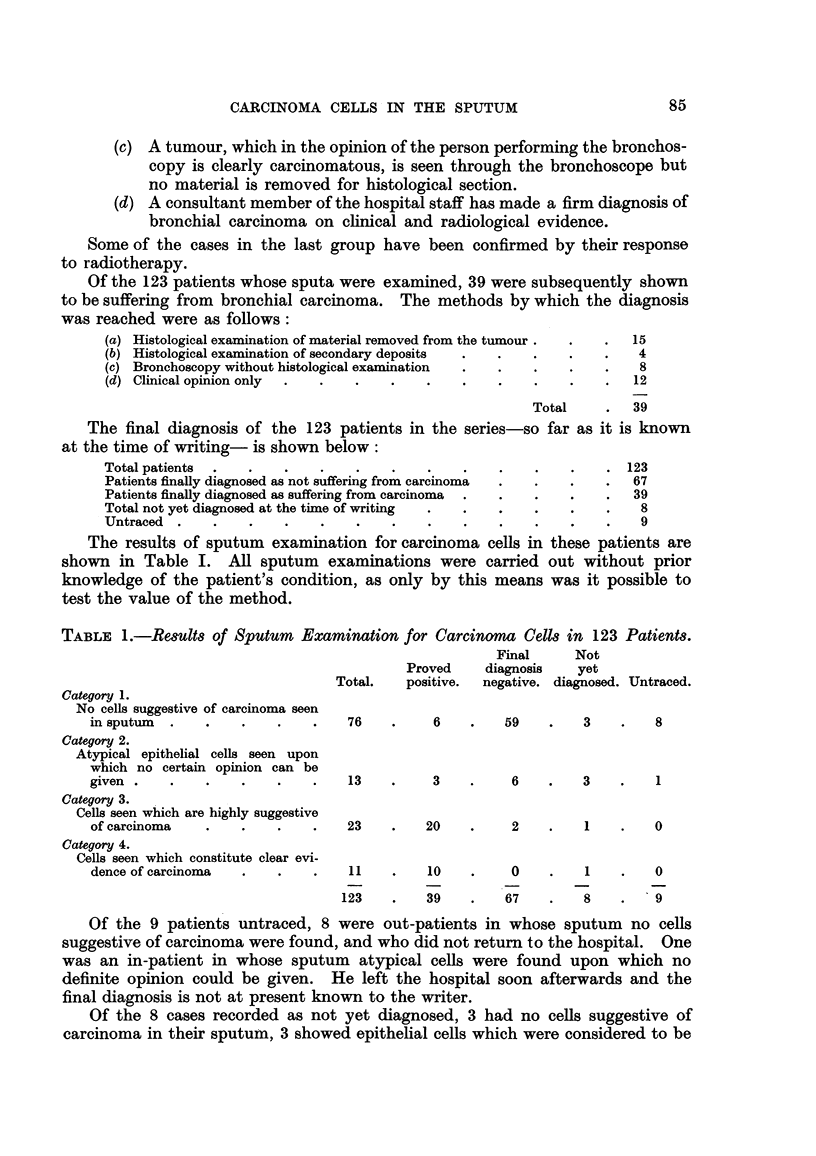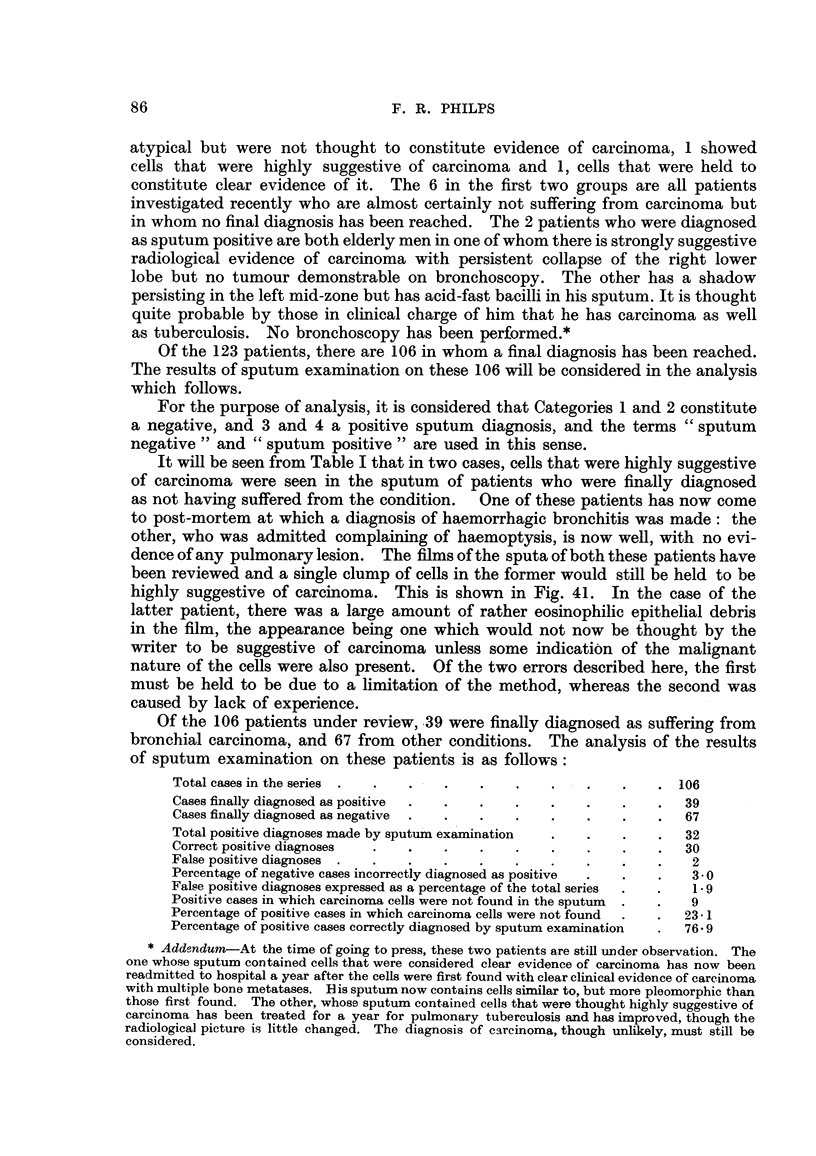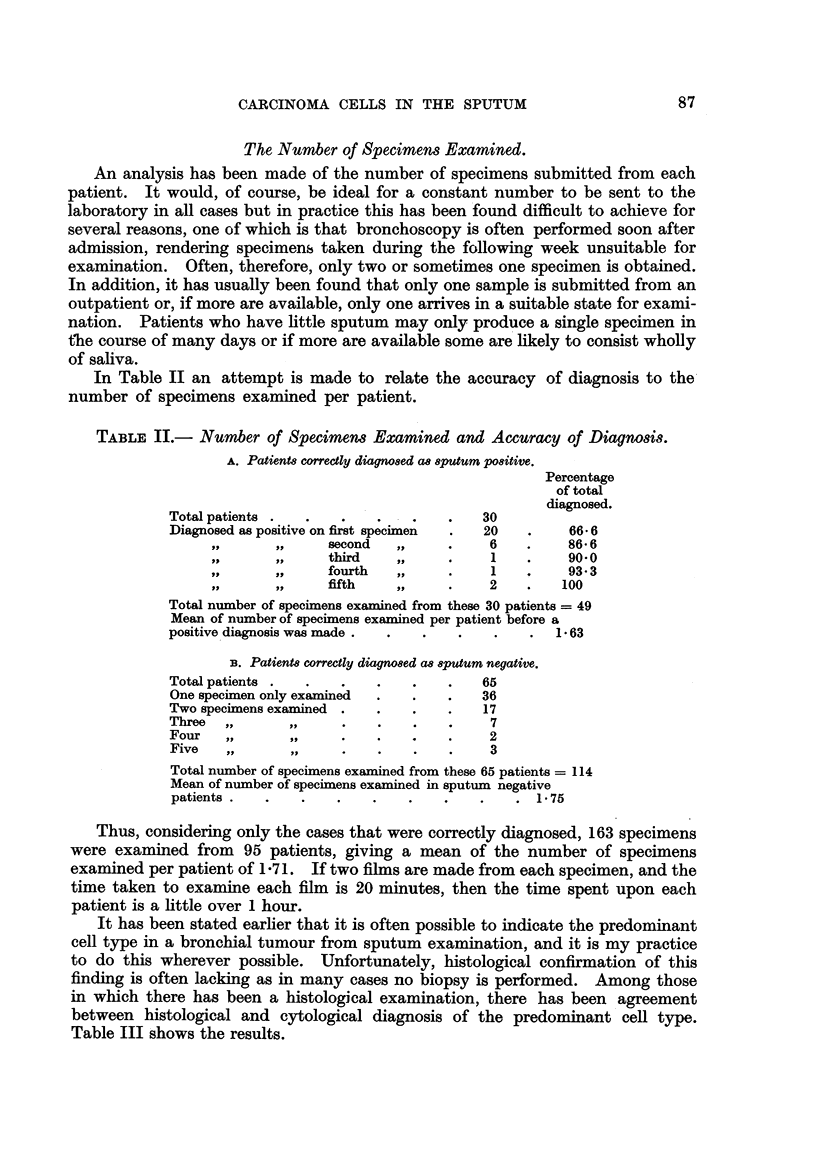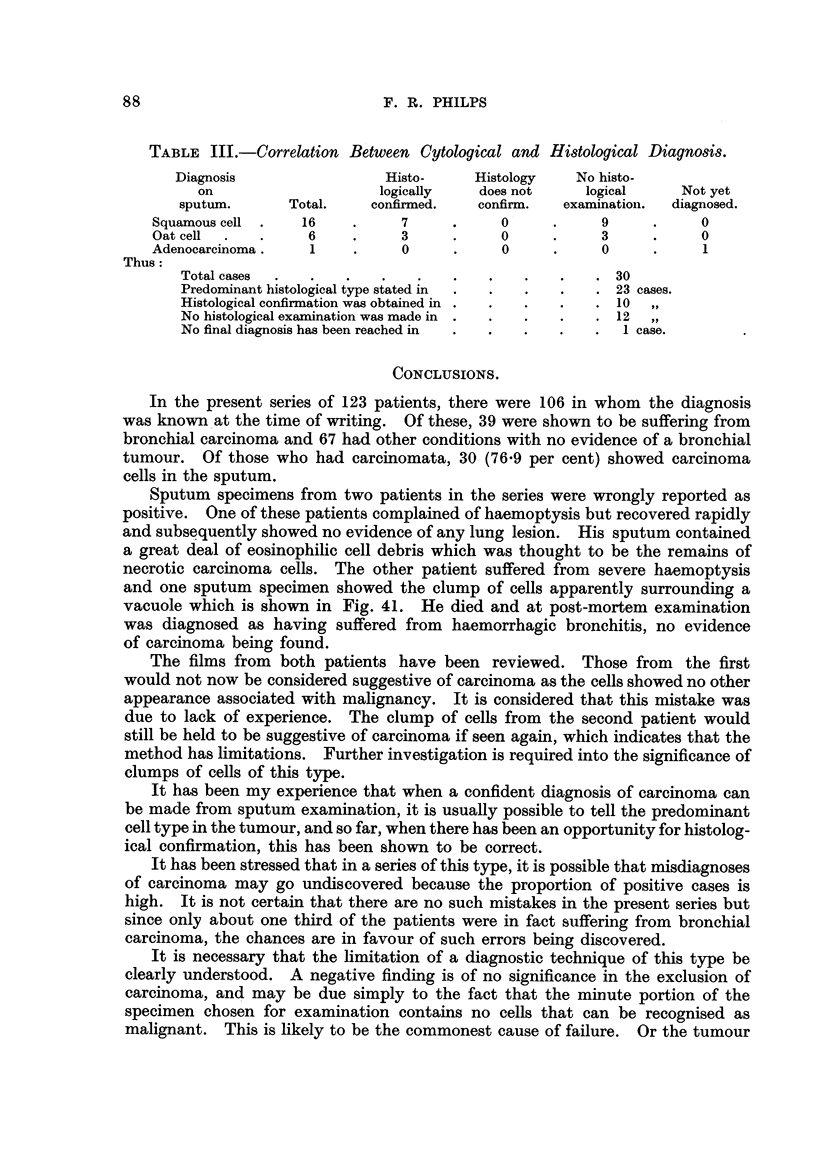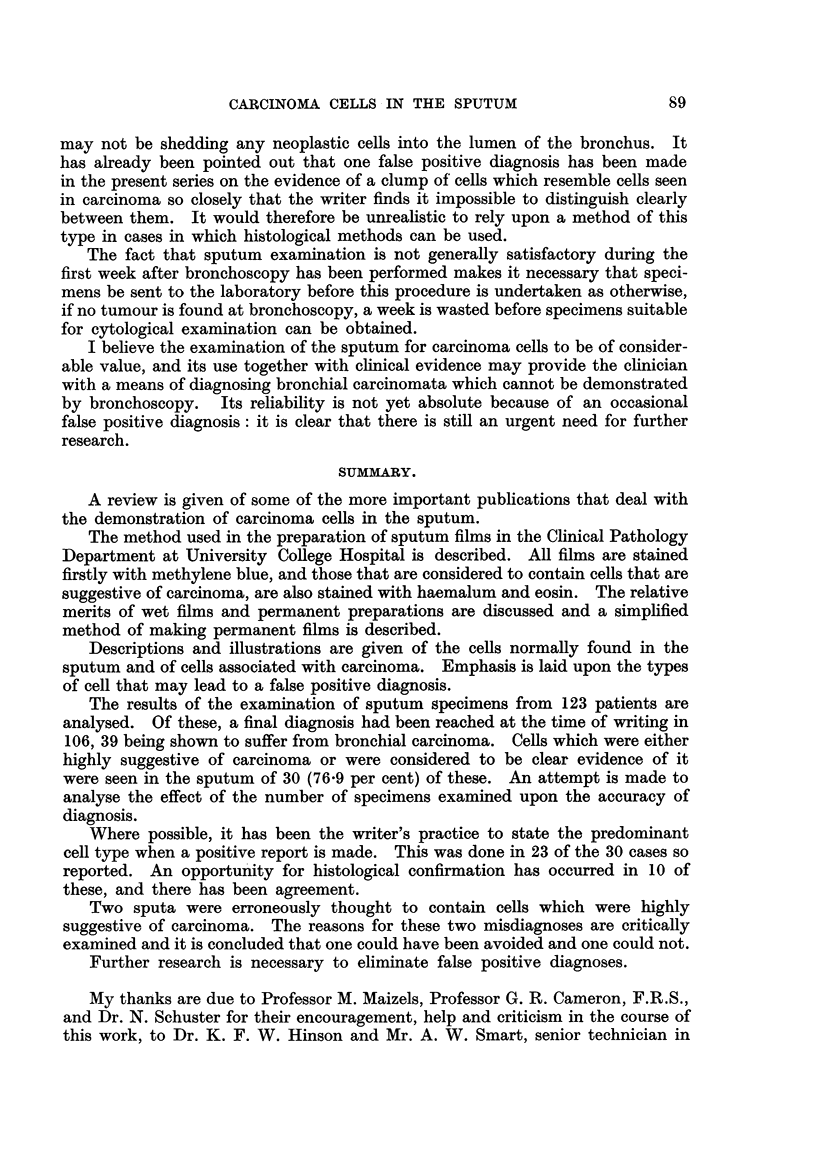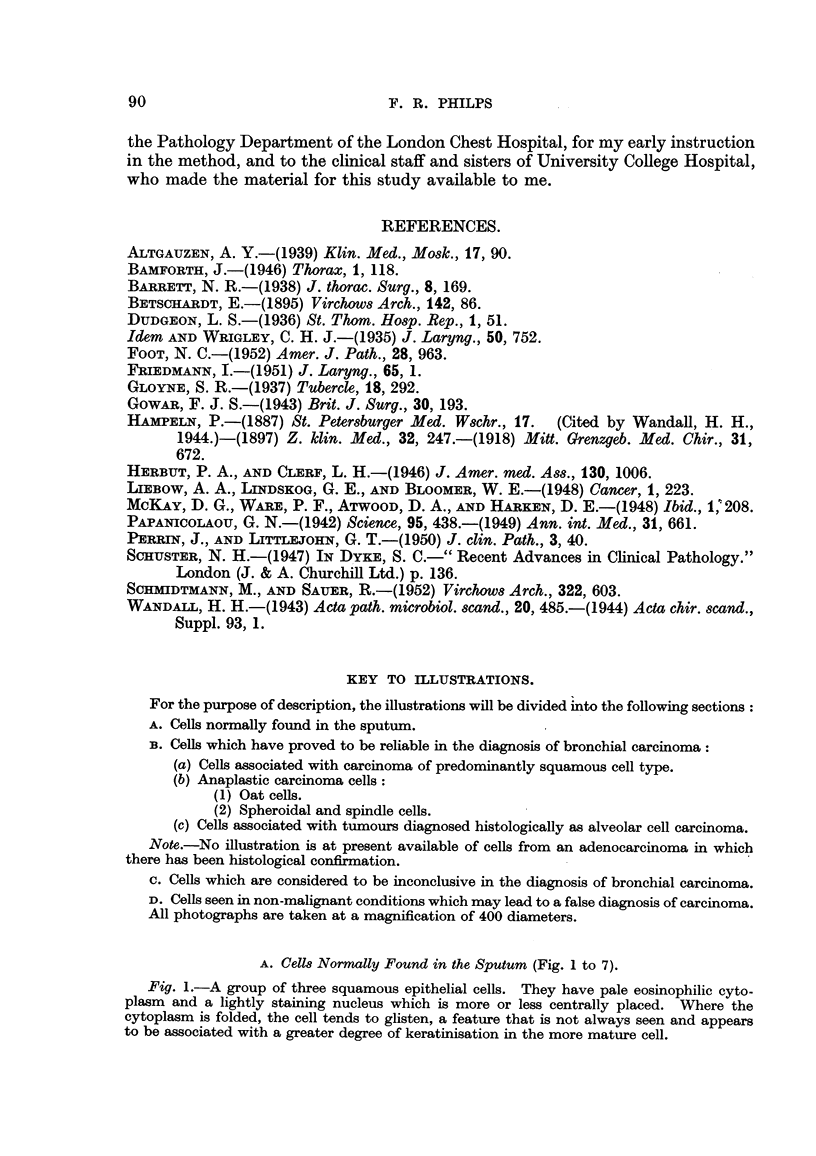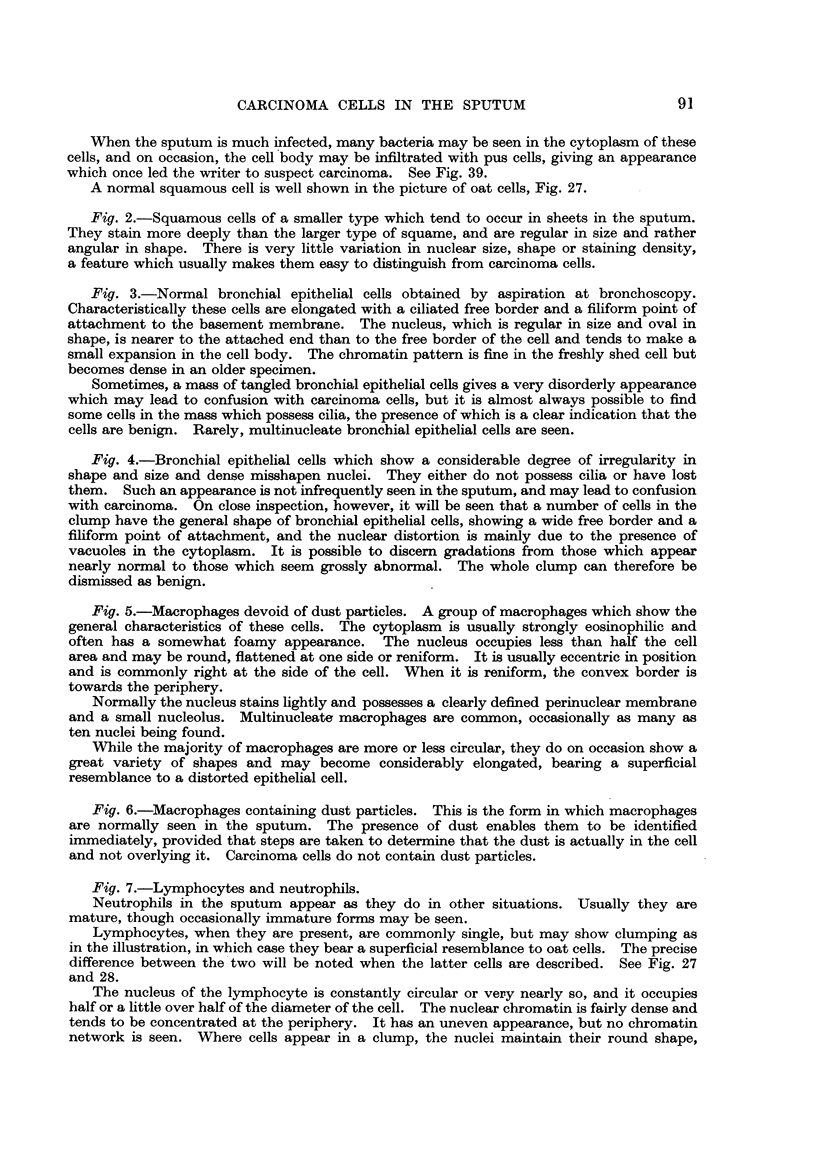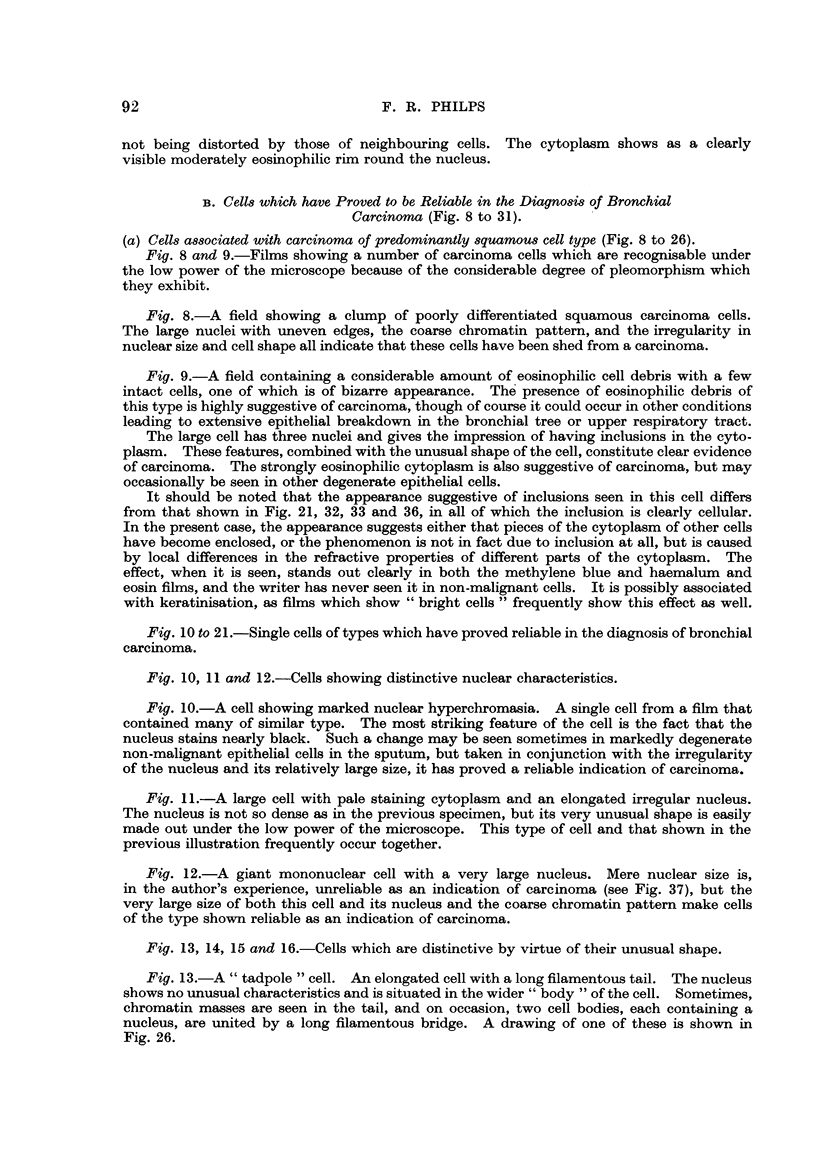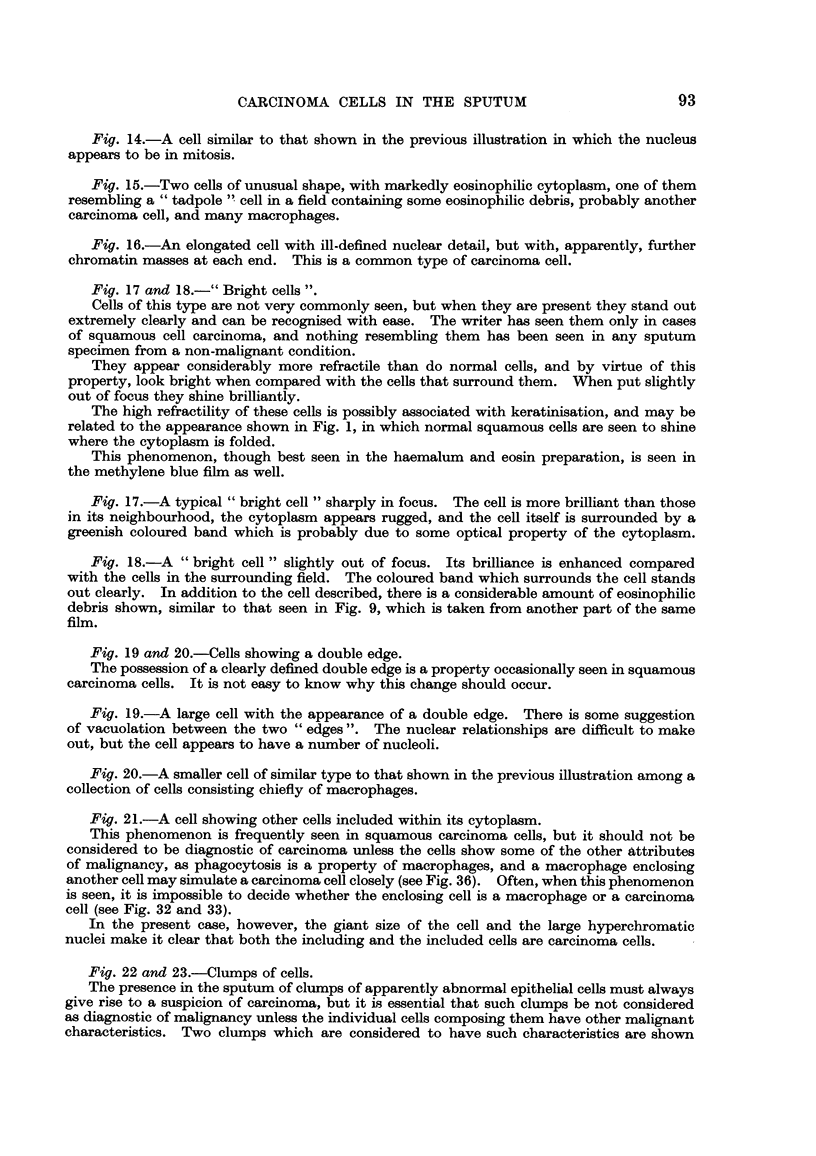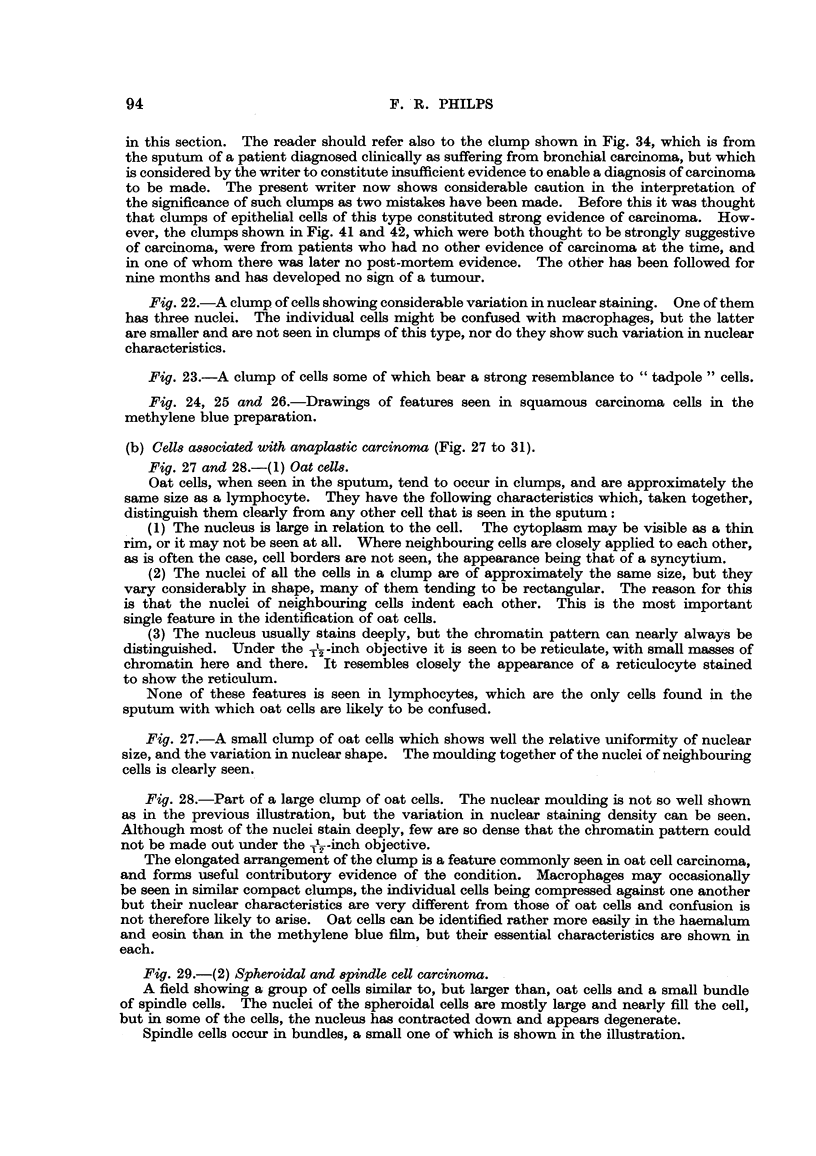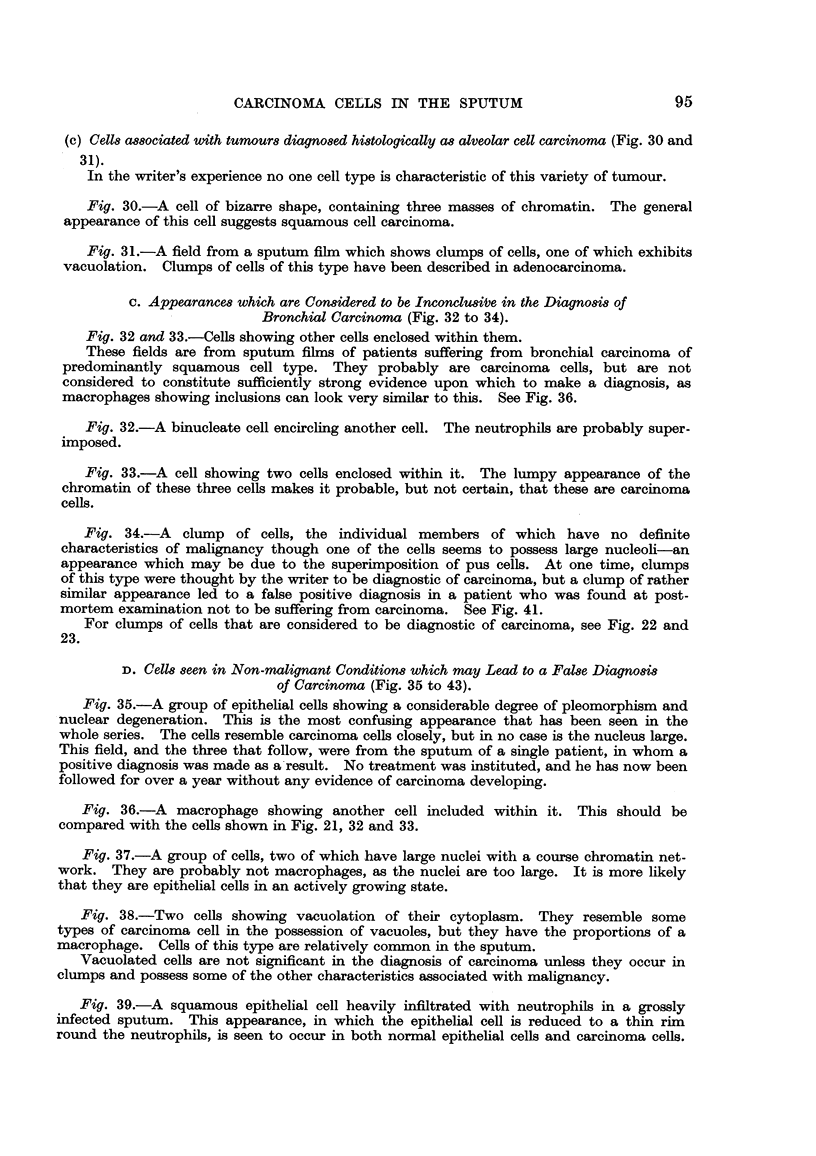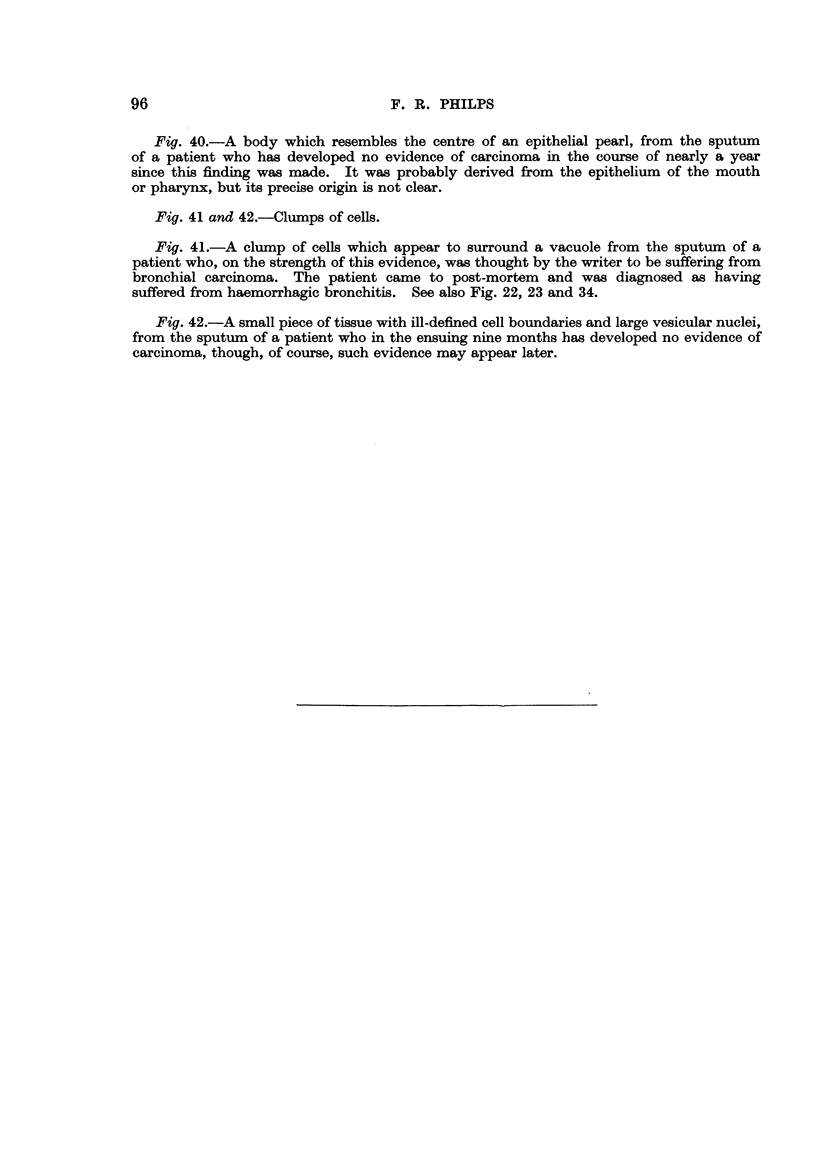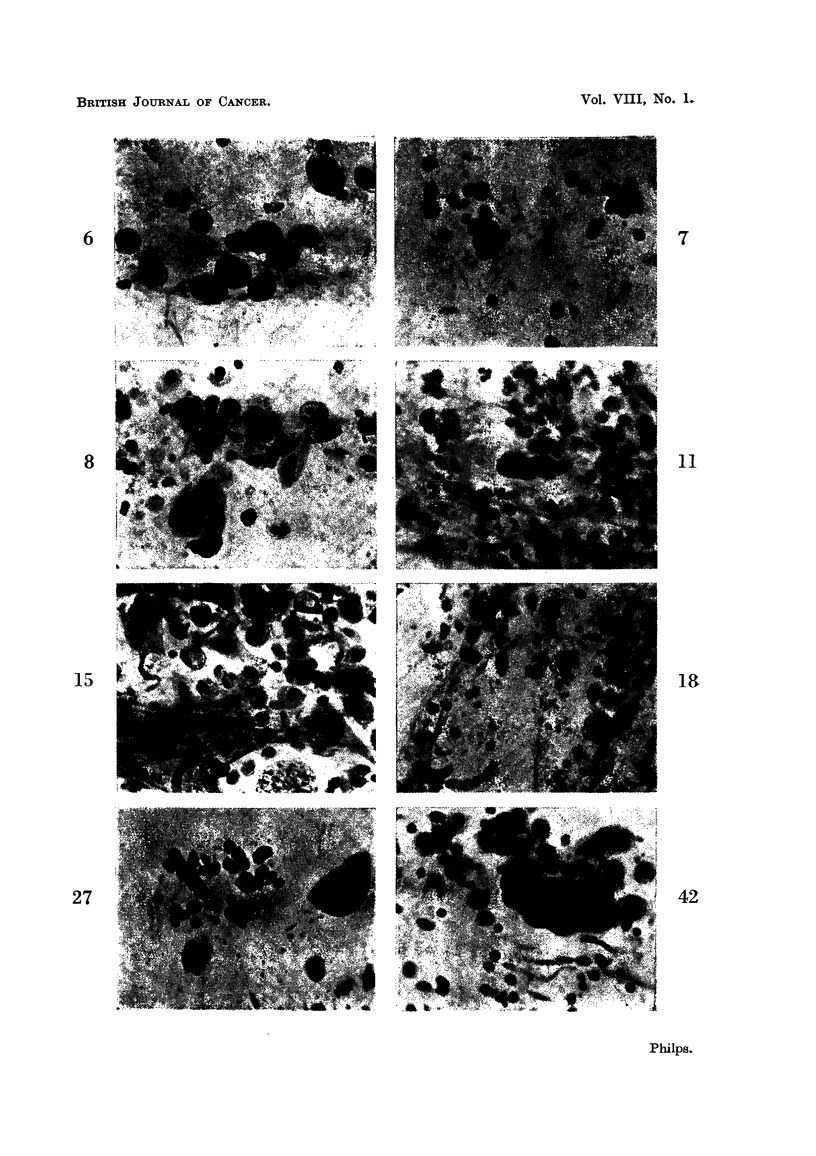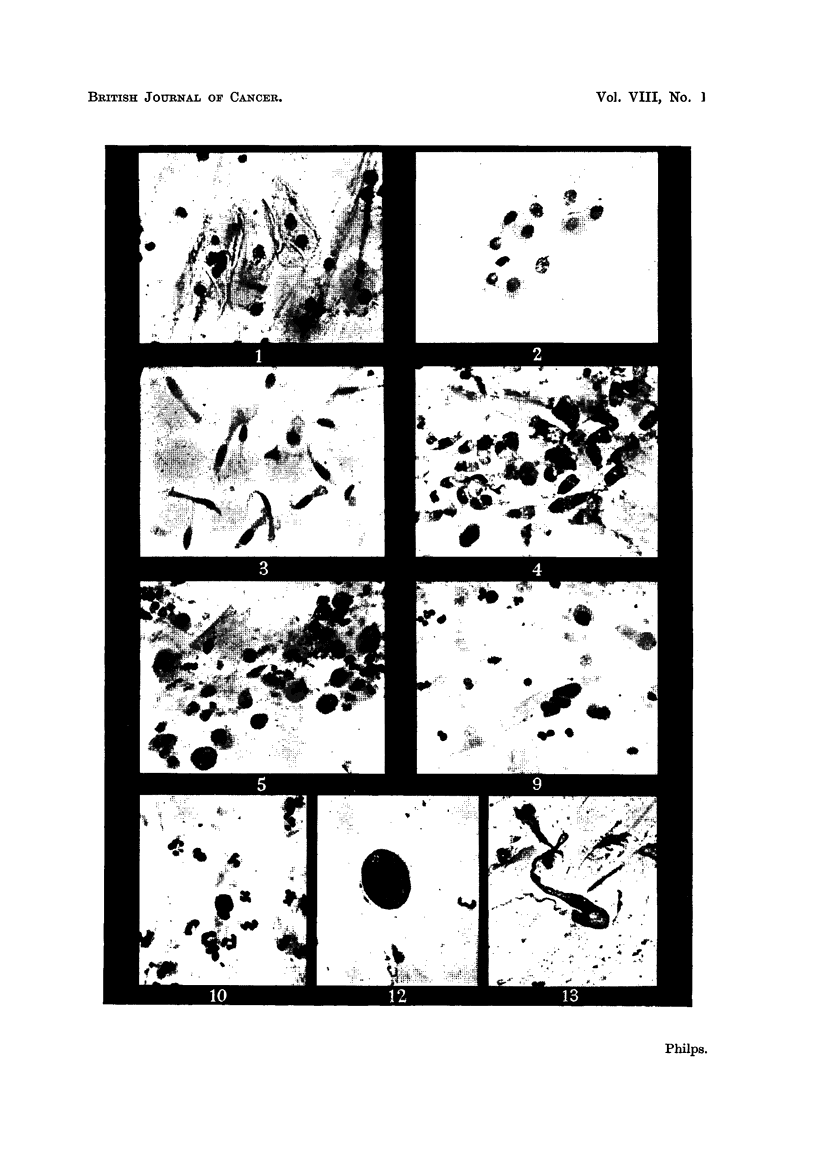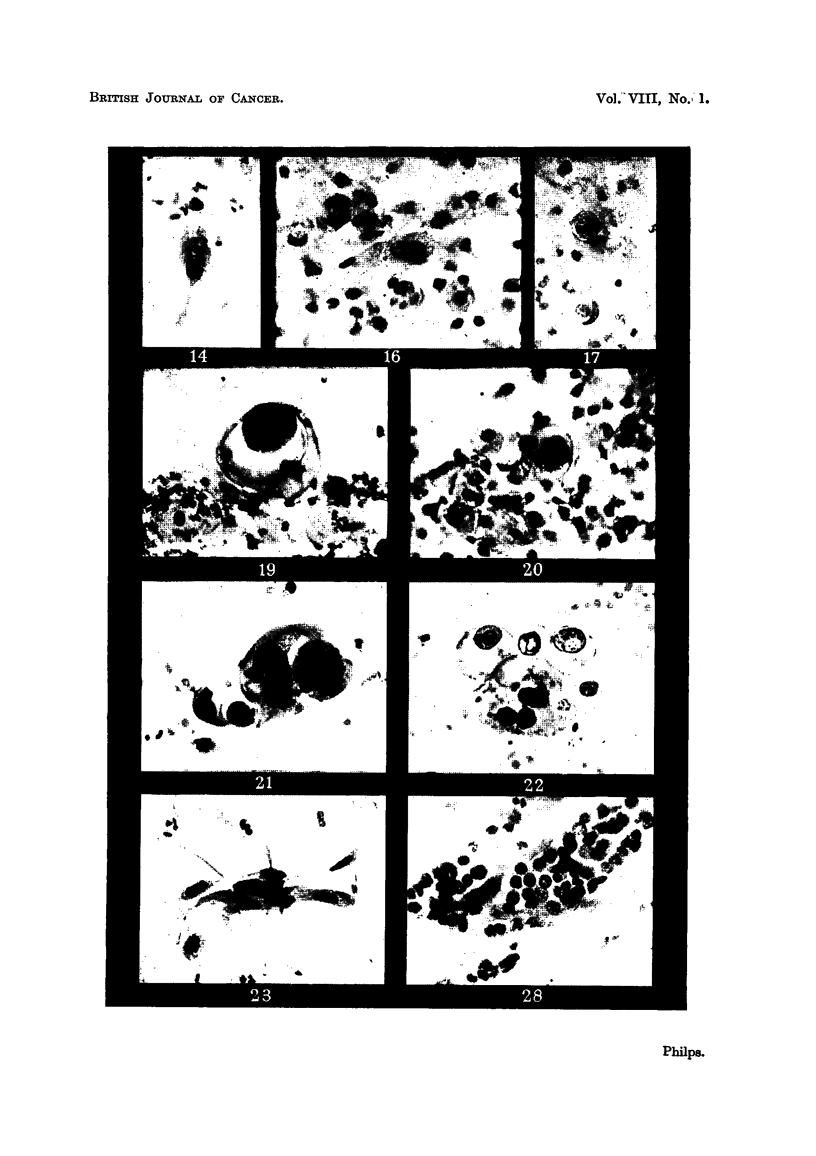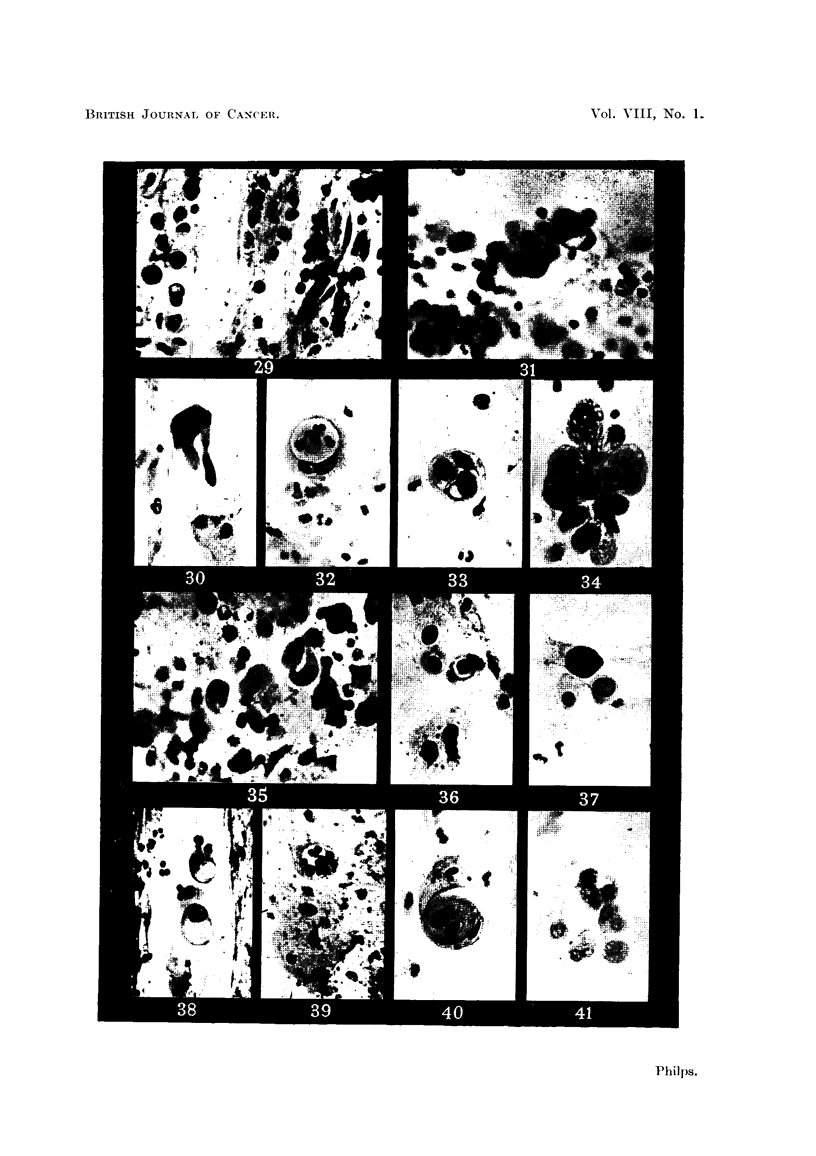# The Identification of Carcinoma Cells in the Sputum

**DOI:** 10.1038/bjc.1954.6

**Published:** 1954-03

**Authors:** F. R. Philps

## Abstract

**Images:**


### THE IDENTIFICATION OF CARCINOMA CELLS IN THE SPUTUM.

F. R. PHILPS.

*From the Department of Clinical Pathology, University College Hospital.*

Received for publication December 5, 1953.

OWING to recent improvements in surgical technique the chances of successful intervention in bronchial carcinoma have much improved, and the need for early diagnosis has correspondingly increased. The early recognition of malignancy, however, may be very difficult and it is hoped that the method described in this paper may be a help towards diagnosis. It does not replace histological examination of material removed at bronchoscopy, but when no tumour can be seen through the bronchoscope, or when histological examination reveals no tumour tissue, it can be of great value.

When there is some contra-indication to bronchoscopy, the demonstration of carcinoma cells in the sputum may be the sole means of establishing a diagnosis.

The demonstration of atypical epithelial cells in the sputum of a patient suffering from bronchial carcinoma is not new. Hampeln in 1887 described a case in which such cells were found. The same writer (1897) and Betschardt (1895) again refer to the occurrence of neoplastic cells in the sputum of patients with bronchial carcinoma. Hampeln in 1918 reported a series of 75 patients suffering from bronchial carcinoma, in 25 of whom the sputum was examined for carcinoma cells. A positive result was obtained in 13 of the 25 cases.

In 1935 Dudgeon and Wrigley published details of a simple staining method that could be used on sputum films, in which they employed haemalum and eosin. They also described some varieties of neoplastic cell found in the sputum. Subsequent papers (Dudgeon, 1936; Barrett, 1938) enlarged upon the original work. At about the same time, Gloyne (1937) described the cytology of sputum with special reference to neoplastic cells. Since then, papers on the subject have been published in a number of countries, including Britain (Gowar, 1943; Bamforth, 1946; Schuster, 1947; Perrin and Littlejohn, 1950), America (Herbut and Clerf, 1946; McKay, Ware, Atwood and Harken, 1948; Liebow, Lindskog and Bloomer, 1948; Papanicolaou, 1949; Foot, 1952), Germany (Schmidtmann and Sauer, 1952), Russia (Altgauzen, 1939) and Denmark (Wandall 1943, 1944). The last-mentioned paper is quite outstanding and forms a valuable work of reference on this subject. It has a full bibliography, particularly of the continental literature.

The proportion of cases of bronchial carcinoma that can be discovered by sputum examination has varied in the hands of different workers. For example, Perrin and Littlejohn (1950) obtained a positive result in 60 per cent whereas Wandall (1944) discovered 84 per cent in this way.

All of the reports to which I have had access have shown that occasionally a false positive diagnosis is made by sputum examination. While it is clear that such mistakes are more likely to be made by an inexperienced worker, there remains the risk that even when the pathologist has considerable experience, errors may still occur. Hence the desirability of histological confirmation of the diagnosis.

The methods described in this paper can be used for examining either sputum or bronchial secretion aspirated through a bronchoscope. Herbut and Clerf (1946) point out that patients with early bronchial carcinoma frequently have no sputum and advocate the examination of bronchial secretion. In my hands the examination of sputum has been more successful and has sometimes been positive when examination of bronchial secretion has yielded a negative result. One virtue of sputum examination is that it causes no discomfort to the patient.

The purpose of this paper is to present photographs and descriptive notes of the cells found in the sputum in both malignant and non-malignant conditions, to indicate those cells which have proved reliable in the diagnosis of bronchial carcinoma, and to stress the difficulties of the method, with special emphasis upon appearances which may lead to a false positive diagnosis. Details are given of the technique used, and the amount of time required for the examination is discussed.

I have been concerned with the diagnosis of bronchial carcinoma only, but carcinoma cells from growths in the upper respiratory tract may also be found in the sputum. For descriptions of these cells, the paper by Friedmann (1951) should be consulted.

In order to gain experience of the appearance of carcinoma cells during the first year of this work, all bronchial carcinomata removed at operation or found at post-mortem were examined by making smears of the cut surface of the tumour. These were stained by both of the methods described in this paper.

### *The Sputum Specimen.*

It is essential that only true sputum, containing a large proportion of bronchial secretion, be examined. The routine is simple for patients who are in hospital. A specimen coughed up in the early morning, before any food or drink is taken, and before the teeth are cleaned, is sent to the laboratory. Very often, although the specimen is produced before breakfast, it is found to contain particles of food material unless the patient has been warned to eat nothing on waking. Specimens that contain particles of food visible to the naked eye should usually be rejected, but when a patient has very little sputum, any specimen that can be obtained should be examined.

Out-patients can be given containers and instructed to bring their sputum in them, but it is important that specimens so produced reach the laboratory as soon as possible, and certainly within 24 hours of being coughed up, otherwise infection and autolysis may destroy the cytological picture.

Specimens sent through the post, particularly in the winter, are occasionally satisfactory, but if long delayed they are useless.

The whole success of sputum examination for carcinoma cells depends in the first instance upon the selection of a suitable piece of the specimen for microscopical examination. In order that it may be clearly seen, the specimen is transferred to a Petri dish and examined against a dark background. A hand-lens may be of assistance.

Areas which contain a large number of macrophages can usually be seen in a transparent specimen as circumscribed, globular, often pigmented masses which can be seen either with the naked eye or with the lens. These globular masses seem never to contain carcinoma cells and are best avoided.

The parts of the specimen which are more likely to show carcinoma cells contain streaks or threads of white or greyish material. In particular, if there are minute threadlike pieces, just visible to the naked eye, which possess sufficient strength to resist breaking when stretched, these should be selected for microscopal examination. This point was made clear to me by Mr. A. W. Smart at the London Chest Hospital, and I have proved its value many times. If the sputum contains pieces of tissue visible to the naked eye, these should be taken for examination, but such fragments are rarely found.

Dudgeon and Wrigley (1935) emphasise the fact that blood-streaked pieces of sputum, when they are present, should be selected. My experience bears this out, but it has been found that deeply blood-stained areas seldom yield a positive result. A mucopurulent piece that is slightly tinged should be taken if it can be found.

If the specimen be frankly and uniformly purulent, then there is no alternative but to take a piece of it at random. Under these circumstances the chance of finding neoplastic cells is not good, but on occasion they may be found, and are often of squamous type.

It has been found that sputum produced during the first few days after the patient has had a bronchoscopy is unsuitable for examination for carcinoma cells. It contains a great deal of cellular material shed from the tracheal lining and the chance of finding neoplastic cells is therefore reduced. I have made it a practice not to examine sputum until a week has elapsed after bronchoscopy.

The most suitable instrument with which to handle sputum is a pair of dissecting forceps that have been sharpened to a point on a grindstone. After a little practice, minute pieces of the specimen can be cut out and manipulated with ease.

The piece of sputum removed from the gross specimen is placed on a slide, thoroughly mixed with the help of the forceps, and half of it transferred to a second slide. One of these is stained with methylene blue (see below), and the other smeared carefully into as even and thin a layer as possible, and placed *while still wet* into fixative preparatory to staining with haemalum and eosin.

It is essential that precautions be taken to avoid disseminating infection from tuberculous sputa. The forceps are therefore kept in 10 per cent lysol when not in use. When they are to be used they are well washed in running water and thoroughly flamed. After use they are put straight back into the lysol without further flaming. A large plug of cotton wool in the bottom of the lysol pot enables them to be cleaned of adherent sputum.

### *Staining Methods.*

A number of staining techniques have been described for the demonstration of carcinoma cells in sputum. None of these is specific for carcinoma cells, but all of them, in the hands of workers experienced in their use, appear to give satisfactory results.

The methods may be divided into two groups : those which result in a permanent preparation and those which only give a temporary wet-film. The first group has the advantage that films may be stored and re-examined in the light of subsequent findings, or compared with histological preparations made from a tumour. Temporary films are quicker to prepare, and it has been stated (Perrin and Littlejohn, 1950) that more sputum may be included in the film, increasing the chance of discovering carcinoma cells. I have not noticed this advantage.

The following permanent methods have been described:

(1) Haematoxylin and eosin. (Gloyne, 1937.)
(2) Haemalum and eosin. (Dudgeon and Wrigley, 1935.)
(3) Haematoxylin, orange G, light green, eosin, bismarck brown. (Papanicolaou, 1942.)

Of the above techniques, the first two have the advantage that the appearances are strictly comparable with those seen in histological films of tumours. The second method has the added merit of simplicity and has been further simplified in the course of the present work.

The following wet-film methods have been described:

(1) Methylene blue. (Schuster, 1947.)
(2) Fuchsin-methylene blue. (Perrin and Littlejohn, 1950.)
(3) Iodine green. (Perrin and Littlejohn, 1950.)

I have had no experience of the last two methods, having found the methylene blue technique satisfactory. The precise technique used differs only in minor detail from that described by Schuster (1947). It was taught to me at the London Chest Hospital, and is as follows:

A 0·5 per cent aqueous solution of methylene blue is used. A small piece of sputum is placed upon the centre of the slide and beside it is put an approximately equal volume of the methylene blue solution. The slide is then warmed by holding it about 8 in. above a bunsen flame, and at the same time the two are rapidly mixed with the forceps, until the excess moisture contributed by the stain has evaporated. The slide should not be overheated, and care should be taken that the mixture is not allowed to dry. The stained sputum is then heaped into a ridge along the middle of the slide, and a cover glass of suitable length immediately put on it and pressed down so that an even thin film is formed. Such films tend to fade after an hour or so, the precise time apparently depending upon the amount of infection present in the specimen, but if it is desired that they last longer, this can sometimes be achieved by sealing round the edge of the cover glass with paraffin wax. Schuster (1947) recommends the addition of glycerine to the stain in order that the preparation may keep longer.

All sputa sent for examination are stained first by this technique, which is quick. Any that appear to contain no bronchial secretion or are heavily contaminated with food material can be rejected at once. Those specimens which contain cells which constitute clear evidence of carcinoma, or are strongly suggestive of it, can be prepared as permanent films so that a record is available for future reference. I make a practice of only reporting as positive those specimens which show evidence of carcinoma in both temporary and permanent films. It has been found that the methylene blue method is somewhat more sensitive than that using haemalum and eosin, particularly in the detection of abnormal cells of squamous type.

If cells of equivocal appearance are seen in the temporary film, further preparations are made, if necessary two or three more, until an opinion can be given. Thus, the methylene blue method is used as a rapid method of sorting the specimens, and the permanent film is used for confirmation and as a record in all cases diagnosed as positive or suggestive of carcinoma.

The technique used for making permanent films is a modification of that described by Dudgeon and Wrigley (1935). It is simple and most of the times given are elastic—a point of some importance in a busy laboratory. The fixative is similar to Schaudinn's and consists of equal parts of a saturated solution of mercuric chloride and absolute ethyl alcohol. A few crystals of mercuric chloride are allowed to remain in the bottom of the vessel containing the mixture to saturate any moisture that may be absorbed by the alcohol. The fixative keeps well, and provided that it is kept free from any suspended particles, can be used repeatedly. It has not been found necessary to add acetic acid before use.

The method is as follows:

(1) Fix in the above mixture for 5 to 10 minutes.
(2) Wash in distilled water for about 5 minutes. This time can be greatly exceeded without harm.
(3) Stain in haemalum for precisely 1 minute.
(4) Rinse in distilled water.
(5) Blue in tap water 5 to 10 minutes.
(6) Counterstain in eosin (1 per cent) for 1 minute.
(7) Rinse in distilled water, then in methylated spirit, dehydrate in absolute alcohol, clear in toluol, and mount in D.P.X.

The haemalum used is a modification of Harris haemalum for which I am indebted to Mr. J. H. Bayley. It is prepared in the following manner: 1 g. haematoxylin is dissolved in 5 ml. of absolute alcohol and the solution added to 100 ml. of saturated potassium alum solution. To this mixture is added a small knife point of sodium iodate and 2 ml. of glacial acetic acid. The stain is ready for use in 24 hours and can be used repeatedly for 3 or 4 months. It should be filtered if it shows a deposit.

It is essential that the film be fixed immediately after it has been spread and while it is still wet, nor at any subsequent stage must it be allowed to dry.

It sometimes happens that a specially characteristic cell or clump of cells is seen in the methylene blue film, and it is desired to make a permanent preparation of it. This can frequently be done in the following way. The position of the cells is marked with a diamond on the underside of the slide. The cover glass is slid off in a direction exactly at right angles to the long axis of the slide, an operation which occasionally destroys the preparation but more frequently leaves the film in place though somewhat smeared in a transverse direction. The slide is immediately dropped into the fixative, where it is left for 5 min. The methylene blue becomes purple during fixation. The slide is then put into absolute methyl alcohol, which removes the stain. Films may be left in methyl alcohol for at least 24 hours without deterioration. When required, they are rinsed in distilled water and stained in haemalum in the usual way. This procedure gives remarkably clear well-stained results. The cells can usually be found in line with the mark on the slide, though sometimes a search is necessary.

*Examination of the Slide.*

Dudgeon and Wrigley (1935) advocate the complete examination of six films from each specimen. Undoubtedly this is desirable, but it has been found in the present series that between 20 and 30 minutes are required for the preparation and complete examination of a film and it has been impracticable to examine six films from each specimen. As has been stated, no positive report has been given unless at least two films have been examined, but it has usually been necessary to report negative findings after the examination of one, or not more than two films. This is bound to limit the accuracy of the report, but has proved satisfactory in practice.

At the outset of this work it was considered that a minimum of three specimens should be submitted from each patient but it has frequently been found that only one or two could be obtained. Especially was this the case with out-patients or those with little sputum. Provided that the piece of the specimen from which the film is made has been very carefully selected, preferably by the person making the examination, and that sufficient time is spent in making the examination itself, this does not appear to be a serious limitation. Obviously the fewer films that are made from the specimens submitted from each patient, the greater will be the likelihood of missing carcinoma cells. The optimum number of specimens to be examined from each patient, and the amount of time to be spent upon each specimen are considered in the analysis of results at the end of this paper.

Some authorities, *e.g.*, Dudgeon and Wrigley (1935), advocate that the film be examined in its total area by racking backwards and forwards across it. The writer has found it more profitable to look at it with the naked eye first so that any parts of it that show a streaky appearance may be especially selected for minute examination. It has been found that carcinoma cells tend to occur in streaks and when these are seen they should be searched throughout their entire length and depth.

Normally, films are examined with the $\frac{1}{3}$-inch objective, the $\frac{1}{6}$-inch objective being used only when likely cells are seen with the low power. There is little demand for the $\frac{1}{12}$-inch objective in this work, though it is occasionally useful for examining the chromatin pattern, especially in oat cells. A rather powerful lamp should be used (a 200 watt bulb is satisfactory) so that the condenser of the microscope may be lowered slightly in order to obtain maximum resolution and contrast. Owing to the fatigue involved, a binocular microscope is especially useful in this work.

I have found it desirable to make a sketch of every cell or cell-type on which a positive diagnosis is made, and of every appearance that gives rise to suspicion of carcinoma, even though it does not lead to a positive diagnosis. In this way, information has been gained regarding the cell-types that are indicative or strongly suggestive of carcinoma. These sketches are particularly necessary in the case of the methylene blue film, of which there is otherwise no permanent record.

*Cells Normally Found in the Sputum.*

1. *Epithelial cells.*

(a) *Squamous cells.*—Squamous epithelial cells that occur in the sputum have usually been shed from the mouth or pharynx. Two types are seen. The commoner sort are typical large squames, roughly polygonal in shape, and from 30 to 100 microns in diameter. The cytoplasm is clear if the cell is not infected, but it frequently contains large numbers of bacteria. It stains pale greenish blue or green with methylene blue, and pink with eosin. In haemalum and eosin preparations, the cell sometimes appears to show folds, and where it is folded, it may glisten (Fig. 1). The nucleus is about 10 microns in diameter, is usually central in position, and in the well-preserved specimen stains lightly with haemalum, showing a fine chromatin pattern. If the cell is degenerate, the nucleus usually stains more darkly. The nuclear border is smooth and devoid of wrinkling or irregularity.

Sometimes, particularly in infected sputum that has stood on the bench for some time, squamous cells are seen which are infiltrated with neutrophil leucocytes. A cell may be so heavily infiltrated that all that is seen is a thin membrane surrounding a mass of leucocytes. This phenomenon may also occur in carcinoma cells and can be a serious source of confusion. Fig. 39 shows such a cell from a patient in whom there was no evidence of carcinoma.

Less commonly, a smaller type of squamous cell is seen, which stains more deeply than that just described. It is about 20 microns in diameter, and usually occurs as part of a small sheet of epithelium in the sputum. The cytoplasm stains pure blue (as opposed to green) with methylene blue, or fairly deep pink with eosin. The outline of the cell is frequently angular—a feature that is evident when such cells are seen lying singly. The nucleus is approximately the same size as that of the larger type of squamous cell, but the chromatin network is somewhat coarser (Fig. 2).

(b) *Bronchial epithelial cells.*— Bronchial epithelial cells, as seen in the sputum, are narrow cells about 75 to 100 microns in length which frequently show a ciliated border at one end, and are pointed at the other. The cytoplasm stains blue with methylene blue and pink with eosin. The nucleus is oval, has a regular outline, and fills the width of the cell, or even expands it a little. It occupies a more or less central position (Fig. 3). These cells, when seen singly in a good state of preservation, present no difficulty, but when they are seen in a mass, as is often the case, they may give a disorderly appearance. In this event, the regularity in size, shape and staining character of the nuclei is a clear guide to their innocence. Rarely, multinucleate bronchial epithelial cells are seen.

Sometimes groups of bronchial epithelial cells are seen which differ from those described above. They are disorderly, show no cilia and have a vacuolated cytoplasm, the vacuoles displacing and distorting the nucleus so that it appears crescentic in shape (Fig. 4). Such cells may be thought to be suggestive of malignancy, but close examination will show that among them are cells which resemble a bronchial epithelial cell closely, except for the possession of cilia, and all degrees of distortion may be discerned between these and those that appear grossly abnormal. In addition, even though the nuclei of these cells are distorted, they are all of about the same size—a feature uncommon in groups of carcinoma cells.

With the exception noted above, normal epithelial cells found in the sputum have the following characteristics :

1. The nucleus is regular in shape, size and staining properties when compared with those of cells of similar type nearby.

2. The chromatin pattern is finer than is usually seen in carcinoma cells in which the chromatin is frequently lumpy and often more concentrated at the periphery of the nucleus.

3. The nucleus in the normal epithelial cell is usually small in relation to the size of the cell. Frequently, though not invariably, it is large in the carcinoma cell (Eig. 8, 12, 21).

4. The perinuclear membrane of the normal epithelial cell is usually smooth and even. In carcinoma cells, particularly of squamous type, it is often wrinkled and irregular.

5. Hyperchromatic nuclei are only seen in non-malignant epithelial cells when these are markedly degenerate. Hyperchromasia is frequently seen in carcinoma cells—indeed, it is one of the characteristics by which they are most commonly recognised.

6. Normal epithelial cells tend to be regular in shape, whereas carcinoma cells, particularly those of squamous type, show an irregularity which is best appreciated when many are seen together in the low-power field. Many squamous carcinoma cells have bizarre shapes (Fig. 9, 13, 14, 15).

7. The cytoplasm of normal epithelial cells is moderately eosinophilic when stained with haemalum and eosin. Some carcinoma cells, particularly those of squamous type, are markedly eosinophilic (Fig. 15). A similar difference may be seen in the methylene blue preparation, when carcinoma cells frequently appear more green than normal epithelial cells. This is not, however, diagnostic, as degenerate non-malignant epithelial cells may show the same staining properties.

2. *Macrophages.*

Macrophages are constantly found in the sputum in large or small numbers, and are sometimes rather difficult to distinguish from carcinoma cells.

Many macrophages contain dust particles, which appear as black or brown granules in the haemalum and eosin-stained preparation, and as black, greenish, or purple granules in a film stained with methylene blue. Sometimes in a film stained by the latter method, the macrophages are seen to contain a large number of highly refractile fat droplets. The presence of dust particles in a cell enables it to be identified as a macrophage, but confusion may arise through the presence of free dust particles overlying a cell and giving the impression of being contained within it.

Macrophages vary a great deal in size. Normally they are from 10 to 50 microns in diameter, but, exceptionally, large forms occur. As a general rule, they are more or less circular in outline, but this feature is by no means constant and they assume a variety of shapes. Spindle shaped and crescentic forms are not uncommon, and occasionally they may assume a tadpole-like shape, and may thus be confused with some types of carcinoma cell if shape alone be relied upon for identification. Usually the cytoplasm is eosinophilic when stained with haemalum and eosin, but it may stain somewhat brownish owing to the presence of ingested particles. With methylene blue, it is usually stained blue, but when there is a large amount of ingested material the colour may be anything from green to pinkish purple. When the cytoplasm contains no dust, it has a foamy appearance which is helpful in the identification of these cells.

There is no constant number of nuclei in a macrophage. Mononuclear forms are most frequently seen, but two nuclei are not uncommon, and cells are occasionally found which contain five or more.

The characteristics of the individual nucleus of the macrophage are fairly constant and are a considerable help in their identification. The nucleus is about 10 microns in diameter and is sometimes circular in outline but is more often flattened along one side or even reniform. It is normally eccentric and often touches the periphery of the cell with the convex border to the outside. Almost always it occupies considerably less than half of the cell area.

The nucleoplasm is not well shown in the methylene blue preparation, but in the film stained with haemalum and eosin it is seen to be fairly clear, with an obvious perinuclear membrane and usually a single small nucleolus. Fig. 5 and 6 show macrophages. Those in Fig. 6 contain dust particles.

3. *Lymphocytes.*

Lymphocytes occur either singly or in small clumps, but are not often found in large numbers in the sputum. They are about 10 microns in diameter and are usually of the small type with a nucleus which occupies the major part of the cell area. The cytoplasm stains blue with methylene blue, and pink with eosin. The nucleus stains rather deeply and may show some unevenness in the distribution of the chromatin. It is circular in shape and the nuclei of neighbouring cells in a clump maintain their shape and do not indent each other. Cells from oat cell carcinomata, when seen in the sputum, are similar in size to lymphocytes, but differ from them in the following ways :

(*a*) The nucleus of an oat cell is larger in relation to the cell area than that of a lymphocyte. The cytoplasm of an oat cell appears as only a thin rim round the nucleus, or it may not be visible at all.

(*b*) The chromatin of an oat cell sometimes stains more deeply than that of a lymphocyte, and when seen under the $\frac{1}{12}$-inch objective, shows as a reticulum as opposed to the rather lumpy chromatin of the lymphocyte.

(*c*) Oat cells nearly always appear in clumps ; lymphocytes seldom do. Frequently, oat cells are so closely opposed to each other that they appear to form a syncytium. The individual cell borders of lymphocytes are always seen.

(*d*) The individual nuclei of oat cells, though usually fairly constant in size, vary considerably in shape. It will be seen that neighbouring nuclei compress and indent each other. Lymphocytes do not show this feature. The only normal cell found in the sputum which occasionally shows this compression effect is the macrophage, which is unlikely to be confused with an oat cell.

Lymphocytes are shown in Fig. 7 ; oat cells in Fig. 27 and 28.

4. *Granulocytes.*

(a) *Neutrophils.*—Neutrophil leucocytes are commonly seen in the sputum, and appear as they do in other situations. Quite frequently the cytoplasm is lost, but in this event the nucleus is easily recognisable.

(b) *Eosinophils.*—Eosinophil leucocytes are found in the sputum in a number of circumstances. In the preparation stained with haemalum and eosin they are always easily recognisable on account of the brilliant pink granules in the cytoplasm.

Apart from the cellular components of sputum, corpora amylacea are commonly found. They are circular bodies, from 10 to 20 microns in diameter. They stain deep blue with methylene blue, and pink, yellowish or brown with eosin. Almost always their non-cellular nature is clear: they may show concentric rings, radial streaking or fissures, or they may be structureless. Very occasionally, a dark irregular body is seen at the centre, which could possibly be confused with a nucleus. This, however, should not happen if the possibility be borne in mind.

Very rarely one of these bodies may be taken up by a macrophage, in which case the risk of confusion with a carcinoma cell is very real if insufficient attention be paid to detail. The body resembles a large hyperchromatic nucleus with a "cytoplasm" which is the body of the macrophage. The fact that the corpus amylaceum is perfectly circular, and has a characteristic structure which does not resemble the chromatin pattern of a nucleus should make its identity clear, and in addition, the macrophage is often seen to contain dust particles. Confusion should not therefore arise. Fig. 43 shows a drawing of a corpus amylaceum

*Fig.* 43.—A drawing of a cell seen in a methylene blue film. The cell "body" is a macrophage, and the "nucleus" is a corpus amylaceum which has been taken up. This appearance should not lead to difficulty, as the lack of any nuclear details and the concentric rings in the corpus amylaceum make its nature obvious. The writer has seen this appearance only once.

enclosed in this manner seen in preparation stained with methylene blue. When stained with haemalum and eosin the body bears no resemblance to a nucleus.

### *Carcinoma Cells.*

Cells which are exfoliated into the sputum from carcinomata exhibit varying degrees of abnormality, ranging from the grossly abnormal to those which resemble normal cells so closely that no certain opinion upon their malignancy can be given. In describing them, therefore, it is considered necessary to give some indication of the degree of reliance in the diagnosis of malignancy that can be placed upon any cell or cell-type when it is seen in the sputum.

In the illustrations that accompany this paper, cells from the sputum of patients suffering from bronchial carcinoma are divided into two classes, namely those which have been found to be reliable for the diagnosis of carcinoma, and those which resemble cells found in conditions unassociated with malignancy sufficiently closely to be unreliable as evidence of it.

Squamous carcinoma cells not infrequently give rise to this type of difficulty. They are highly pleomorphic, and cells from one growth may appear nearly normal or may be so abnormal that there is no difficulty in recognising them for what they are. Cells from anaplastic and adenocarcinoma are less likely to cause confusion for though they may bear a superficial resemblance to cells normally found in the sputum, they can be distinguished with reasonable certainty by an observer practised in the finer points of differentiation.

The difficulty with squamous cells is not so great as may be imagined, as their very pleomorphism tends to reveal them for what they are. Although some of the cells found may closely resemble normal cells, it is usually not difficult to find grossly abnormal cells among them upon which a diagnosis may be made. It is emphasised that a diagnosis is very rarely made upon the appearance of just one or two abnormal cells, for even if these be scanty (and in the early case they may well be), prolonged search will almost always reveal further abnormal cells.

If cells which are suggestive of carcinoma are very scanty in the sputum, a wise plan is to search carefully in the immediate neighbourhood of any such cell that is found, as there is a good chance that other cells may be nearby. I have often found that by adopting this plan, cells which have been considered to be no more than suggestive of carcinoma have enabled me to find other more convincing evidence of growth.

Although it is unwise to be unduly dogmatic in typing bronchial carcinomata, it has been my practice to report the predominant cell type so far as this can be determined. It has been found that this can be done with considerable accuracy. In this section of the paper, therefore, the appearances will be described according to the type of tumour in which they are found, namely:

1. Cells associated with carcinoma of predominantly squamous cell type.
2. Cells associated with anaplastic carcinoma:
   (*a*) Oat cell.
   (*b*) Spheroidal and spindle cell.
3. Cells associated with adenocarcinoma.
4. Cells associated with tumours diagnosed histologically as alveolar cell carcinoma.

1. *Cells associated with carcinoma of predominantly squamous cell type.*

There is no single feature that distinguishes squamous carcinoma cells from those of other types, but the following characteristics may be seen:

They are usually large cells, the majority being more than 20 microns in diameter, though if the cell is considerably elongated the smaller diameter may be much less than this (Fig. 13).

They may be seen in a great variety of bizarre shapes. Nearly all types of carcinoma cell (with the exception of oat cells) show a considerable degree of pleomorphism when seen in the sputum, but cells of squamous type exhibit this feature to a more marked degree than the others. If there are many such cells in the film, this pleomorphism is evident under the low power in both methylene blue and haemalum and eosin preparations, and is of considerable help in establishing a diagnosis (Fig. 8, 9).

With regard to the shape of the cells there is no rule that can be followed in their identification, but certain shapes do tend to recur. One of these is the so-called " tadpole cell " (Fig. 13, 14) which consists of a cell body with a long filamentous tail, the body usually containing the nucleus, though clumps of nuclear material may be seen on occasion in other parts of the cell as well. It has been mentioned that very rarely macrophages may assume this shape, but they can usually be recognised from their nuclear characteristics and the presence of dust particles within the cytoplasm. Another shape that is sometimes seen and which, in my opinion, is diagnostic of squamous cell carcinoma consists of two cell bodies, each containing a nucleus, united by a long filamentous bridge—as it were a double " tadpole cell " (Fig. 26). Though it is a good rule to suspect

*Fig.* 26.—Two cell bodies united by a filamentous cytoplasmic bridge. This appearance is not commonly seen, but when present it is considered by the writer to be diagnostic of squamous cell carcinoma.

carcinoma whenever considerable pleomorphism is seen in epithelial cells, it should be stressed that a moderate degree may be seen in conditions unassociated with carcinoma. Fig. 35 shows an appearance which was mistakenly thought to be due to carcinoma in a patient who showed no other evidence of a tumour at the time, who has been followed for a year and has developed no sign of the disease.

The staining reaction of the cytoplasm is somewhat variable, but may be of some help in identification. With methylene blue, it frequently stains greener than that of normal cells, but this property is shared by any degenerate epithelial cell, be it from a carcinoma or from a non-malignant condition, so it is in no way diagnostic. The cytoplasm of many squamous carcinoma cells is peculiar in that it appears to be more refractile than that of normal epithelial cells. In the methylene blue preparation, this property shows itself by the presence of a dark, rather emerald green outline to the cell and sometimes also to the nucleus within it. The cytoplasm itself appears lighter and slightly luminous compared with that of the surrounding normal cells.

When stained with haemalum and eosin, the cytoplasm as a general rule stains bright pink, occasionally with a tinge of orange. Frequently, as in the methylene blue film, the cell appears brighter than those round it, and if it is put slightly out of focus it may shine up quite brilliantly. Cells of this type have been named "bright cells " and are in my opinion diagnostic of squamous cell carcinoma. They are shown in Fig. 17 and 18.

Not uncommonly the cytoplasm may show some degree of variation in staining density which can be of considerable assistance in the diagnosis of carcinoma. This is much more clearly seen in the methylene blue preparation, and takes the form of a suggestion of wrinkling which is most evident near the periphery and is concentric with it. Such an appearance may indicate keratinisation. It has proved reliable for the diagnosis of squamous cell carcinoma (Fig. 25).

*Fig.* 25.—Cells from squamous cell carcinomata that give the appearance of wrinkling of the cytoplasm.

Often, squamous carcinoma cells show a series of irregular concentric lines in the cytoplasm, which appear to be minute folds. These may be seen most commonly at the periphery, but they may also occur round the nucleus.

Although they are seen to the best advantage in the methylene blue film, they show in the permanent film as well.

Another feature frequently seen in the methylene blue film is a mass of minute refractile bodies which are usually disposed in a circular manner round the nucleus. On close inspection, the individual particles sometimes appear to be angular in outline rather than circular. They stain with sudan III, and are therefore lipoid in nature. Provided that they are clearly distinguished from the dust particles and oil droplets (which are larger and obviously circular in outline) that are seen in macrophages, these particles are useful contributory evidence of carcinoma (Fig. 24).

The nuclei of squamous carcinoma cells have few constant features. In fact, they show an extreme degree of variability in size, shape and staining density. Usually, the nucleus of a carcinoma cell is larger in relation to the size of the cell than is that of a normal epithelial cell (Fig. 12), but the presence of a large nucleus cannot in itself be considered to be evidence of carcinoma, as other cells in the sputum may occasionally show this feature (Fig. 37.)

Multinucleate carcinoma cells are commonly seen, but the property is not diagnostic of carcinoma as it may be found in macrophages and very occasionally in bronchial epithelial cells.

A spurious effect of multinucleation may sometimes be given by two epithelial cells overlapping each other.

The shape of the nucleus is of considerable help in diagnosis. Often, the nucleus of a squamous carcinoma cell is irregular in shape and frequently shows a very uneven edge. This wrinkling and irregularity of the perinuclear membrane is occasionally seen in degenerate epithelial cells in non-malignant conditions, but when it is seen in conjunction with a large nucleus, it is practically diagnostic of squamous cell carcinoma.

*Fig.* 24.—Cells showing collections of minute refractile particles in the cytoplasm.

These particles are transparent and tend to occur most commonly in a circular arrangement round the nucleus, but they may be seen in any part of the cell. They give the impression of being angular, but they are so minute that it is difficult to be certain of this. They can be shown to take up sudan III, and are therefore of lipoid nature. They are almost certainly associated with cell degeneration and consequently are not a constant feature of carcinoma cells. They are not seen in the haemalum and eosin preparation. Normal squamous epithelial cells not uncommonly show similar particles in the neighbourhood of the nucleus, but in the writer's experience, these are not seen in such large numbers as in carcinoma cells, nor are they concentrated so definitely in one portion of the cell.

These particles must be clearly distinguished from the larger, usually black, dust particles and from the large circular fat droplets that are commonly seen in macrophages. Dust particles usually present no difficulty owing to their larger size, dark colour and the fact that they are evenly distributed throughout the cell. Fat droplets are usually evenly distributed, and appear globular.

The six drawings, and those in Fig. 25, indicate the variations in nuclear staining density and character that may be seen in preparations made by this method.

As a general rule, the nucleus stains densely, and not infrequently it appears nearly black (Fig. 10, 11). This extreme degree of hyperchromasia may also be seen on occasion in any degenerate epithelial cell, and is not, in itself, diagnostic (Fig. 35). Where the chromatin pattern is visible, it nearly always appears coarse and lumpy, as opposed to the much finer pattern of normal epithelial cells (Fig. 8, 12). These observations apply to both the methylene blue and the haemalum and eosin preparations. Occasionally, the nucleus hardly stains at all, and can be seen only as a faint outline. The presence of a nucleus which stains in this manner, particularly if it appears rather larger than normal, is very suggestive of carcinoma.

Nucleoli are occasionally seen in squamous carcinoma cells, and while they are somewhat suggestive of carcinoma, if they are large in size or multiple, I do not place any reliance upon them in the diagnosis of carcinoma of this type.

Mitosis is rarely seen in carcinoma cells in the sputum, and when it does occur, there is usually plenty of additional evidence that the cells are malignant. The presence of mitoses in epithelial cells does not imply malignancy, as it may occur in any condition in which there is a considerable amount of epithelial regeneration.

In addition to the features mentioned above, a number of other characteristics may be seen which are of considerable help in the diagnosis of carcinoma of squamous cell type. They are as follows:

1. The cell may show a definite double edge, as shown in Fig. 19 and 20. This appearance, if it is as clearly marked as in the specimens shown in the photographs, is considered to be diagnostic of squamous carcinoma.

2. Cells may be seen to be included one within another. Such an appearance must, however, be interpreted with great caution, as phagocytosis of cells is not exclusively a property of carcinoma cells and is sometimes seen in macrophages (Fig. 36). Often therefore such cells must be considered to be inconclusive as evidence of carcinoma as it is impossible for the examiner to be sure that the including cell is not a macrophage (see Fig. 32 and 33 which were seen in films of sputum from patients suffering from bronchial carcinoma of squamous cell type). Though the phenomenon of cell inclusion is bound to arouse suspicion of carcinoma, the including cell must show other characteristics associated with malignancy before it can be considered to provide reliable evidence of the condition. Such a cell is shown in Fig. 21.

3. The occurrence of clumps of cells showing nuclear pleomorphism has already been noted. A particular type of clump has been found to occur in both squamous cell and adenocarcinoma, and though not absolutely diagnostic of malignancy appears to be nearly always associated with it. A clump of this type is shown in Fig. 22. The cells all appear to spring from a central point and the clump gives the impression that it may have formed part of a polypoid mass that has broken off into the sputum. If this is correct then any condition associated with papilliferous projections into a bronchus could give rise to such clumps and they might be expected in the sputum in bronchiectasis. I have not seen such clumps in bronchiectatic sputum, but a clump of this type has, nevertheless, led to a mistake. A single group of cells (Fig. 41) was found in the sputum of a man complaining of haemoptysis, and was reported as being highly suggestive of carcinoma. No treatment was undertaken and the patient subsequently died. At post-mortem examination, it was found that he had suffered from haemorrhagic bronchitis. It is clear, therefore, that an appearance of this type should not lead to a diagnosis of carcinoma unless the individual cells show other characteristics associated with malignancy.

*Squamous metaplasia.*—The problem of squamous metaplasia in the bronchial epithelium, and the possibility that this condition may be confused with squamous cell carcinoma from sputum examination should be mentioned here.

Squamous metaplasia unassociated with carcinoma is probably uncommon, though its precise incidence is unknown. It has been demonstrated in some cases of bronchiectasis and may occur in other chronic infective conditions.

In the presence of such a change, cells from the metaplastic area would probably be seen in the sputum, but their morphology is not clearly known.

Occasionally, round cells of squamous type which have the peculiar glistening cytoplasm that one associates with keratinisation are seen in the methylene blue preparation. These cells differ from carcinoma cells in that their nuclei are regular in size and shape, though they are often somewhat large in relation to the size of the cell. When stained with haemalum and eosin the cells appear normal except for their round shape. They do not suggest carcinoma in the latter preparation, though they may give rise to suspicion in the methylene blue film. It appears that cells of this type are young squamous cells which may have been derived from the mouth or pharynx or may possibly have come from a metaplastic area in a bronchus. From a practical standpoint, the fact that these cells appear abnormal in the methylene blue film, but practically normal in that stained with haemalum and eosin is of some importance and indicates the necessity of confirming with a permanent preparation all positive findings made on the methylene blue film.

2. *Cells associated with anaplastic carcinoma*

(*a*) *Oat cells.*—The characteristics of oat cells have been briefly described already in the section outlining the difference between them and lymphocytes (see p. 75).

They are small cells, 10 to 12 microns in diameter, and frequently occur in compact clumps in the sputum. Although the nuclear characteristics tend to be somewhat ill-defined in the methylene blue preparation, this clumping should always give rise to a suspicion of oat cell carcinoma.

Frequently the clumps tend to be elongated. Mention has already been made of the fact that macrophages may also be seen in clumps, and these also may be elongated, but here the individual cells differ considerably, and can be recognised by the relative sizes of the nuclei, even though the macrophages contain no dust particles. In the methylene blue preparation, oat cells usually stain much more densely than do macrophages.

When stained with haemalum and eosin, oat cells tend to be fairly constant in their characteristics. The cytoplasm is scanty, forming at the most a thin rim round the nucleus, which nearly fills the cell. In many cells, no cytoplasm at all is seen : in others, when the clump is examined under the $\frac{1}{12}$-inch objective, cell borders cannot be defined, the cells appearing to form a syncytium. The cytoplasm when seen stains pink with eosin.

The nucleus stains deeply with haemalum, and is seldom circular as the nuclei of neighbouring cells indent each other, giving the appearance of having been pressed together. This feature is of great importance in the identification of these cells, and in conjunction with the clumping and relatively large nuclear size, appears to be diagnostic and makes the cells easy to recognise. The nuclear chromatin is usually clearly defined when the cell is seen under the $\frac{1}{12}$-inch objective, and is reminiscent of the appearance of the reticulum of a stained reticulocyte. Less commonly, particularly in degenerate cells, the nucleus may be denser and the chromatin network difficult to define. Oat cells are shown in Fig. 27 and 28.

(b) *Spheroidal and spindle cells.*—I have had experience of only one carcinoma of this type which was diagnosed by sputum examination. The sputum in this case contained neoplastic cells arranged in streaks rather than clumps. A typical appearance is shown in Fig. 29. The two types of cell could be clearly distinguished from each other. The round cells were about twice the size of an oat cell, that is, from 20 to 25 microns in diameter, and they showed greater variation than do oat cells. In some, the nucleus was vesicular and nearly filled the cell : in others, it appeared somewhat contracted and stained more deeply, while in the third and commonest type the nucleus was quite small and stained nearly black. Spindle cells were seen in clumps among the streaks of round cells. They were long with a central elongated dense nucleus which filled the width of the cell. The individual cells in the clump showed great variation in cell and nuclear size, which made them clearly recognisable from bronchial epithelial cells, with which they might otherwise have been confused.

3. *Appearances associated with adenocarcinoma.*

Adenocarcinoma has been diagnosed only once in the present series by examination of the sputum, and in this case there has been no histological examination to confirm the finding. Dudgeon and Wrigley (1935) and Wandall (1944) describe the appearances in this type of carcinoma. Typically, the cells appear in clumps and show vacuolation of the cytoplasm, the vacuoles frequently filling the cell and pushing the nucleus to one side, often flattening it against the cell wall.

The appearance shown in Fig. 31, which is from the sputum of a patient diagnosed histologically as suffering from alveolar cell carcinoma, is very similar to that described by the above-mentioned writers in adenocarcinoma.

The presence of cells showing vacuolation is not in itself evidence of carcinoma. The cells shown in Fig. 38 were from the sputum of a patient who showed no evidence of carcinoma, and who, because of the presence of these and similar cells has been followed for a year and has developed no tumour. Therefore, unless the cells showing vacuolation occur in clumps and show other characteristics associated with malignancy, they must be considered to be unreliable as evidence of carcinoma.

Quite frequently, in sputa showing ample evidence of carcinoma of squamous cell type, clumps of cells are seen which surround a single vacuole. Whether this appearance occurs as part of the picture of squamous cell carcinoma, or is an expression of the pleomorphic nature of bronchial carcinoma in general is difficult to decide.

Cells of adenocarcinomatous type might be expected in the sputum of patients with pulmonary metastases from adenocarcinomata elsewhere ; in my experience such a finding is rare.

4. *Appearances associated with tumours diagnosed histologically as alveolar cell carcinoma.*

I have had experience of 4 patients suffering from tumours of this type. Three were diagnosed in the first instance by sputum examination, the tumour being inaccessible to the bronchoscope, and the finding was confirmed after resection. The fourth was a patient in whom there was a recurrence after operation. I have formed the opinion that there is no characteristic cytological appearance in the sputum associated with this type of carcinoma. A wide variety of cells was found in the sputum of these 4 patients, including those which suggested squamous cell carcinoma (Fig. 30), anaplastic carcinoma and adenocarcinoma (Fig 31).

It was possible in all these cases to make a diagnosis of carcinoma from sputum examination, but in no case was it possible to state the type of growth that was present.

*Reporting Results.*

It has been my practice to report the results of sputum examination in four categories, which are as follows :

Category 1. No cells suggestive of carcinoma seen.
,, 2. Atypical epithelial cells seen upon which no certain opinion can be given.
,, 3. Cells seen which are highly suggestive of carcinoma.
,, 4. Cells seen which constitute clear evidence of carcinoma.

Categories 1, 3 and 4 appear from the results to be valuable as an indication of whether the patient has carcinoma, but the value of Category 2, judging from the analysis of results which follows, appears open to question. It will be seen that of the 13 patients whose sputa were reported in this category, 3 had cancer, 6 had no evidence of a tumour, 3 are not yet diagnosed but almost certainly are not suffering from carcinoma, and 1 has not been traced. Thus, among the 13, considering only those in whom a final diagnosis has been reached, there are 3 positives and 6 negatives, and if the probable diagnoses are considered then there are 9 negatives. Such a report gives the clinician no indication of the pathologist's opinion, and is therefore of no assistance to him. It is my intention to stop using this grade and to report specimens in three categories only, those which would have been returned in Category 2 being classed as negative.

*Analysis of Results.*

When interpreting the results of a series of this nature, it is essential to realise that the sputa under examination come from potentially positive cases, as specimens are sent only from patients in whom bronchial carcinoma is suspected. The proportion of positive results, therefore, is greater than it would be for an equivalent number of random specimens and there is a possibility of reporting as positive a specimen from a patient who has in fact a bronchial carcinoma, but whose sputum contains no malignant cells. Clearly, the larger the proportion of actual cases of bronchial carcinoma, the greater is the risk of such undiscovered mistakes. During the course of this work the number of proved positives totalled a little over one third of the whole, so the chance of discovering misdiagnoses was reasonably good.

The present investigation is based upon the results of all sputum examinations for carcinoma cells performed from mid-September 1952 until the end of March 1953, and comprise 123 patients. Before beginning the investigation, I examined the sputa of 165 patients in the course of a year. Approximately 750 films were made from these specimens.

In the present series, a patient is considered to be suffering from bronchial carcinoma if one of the following criteria be satisfied :

(*a*) There is histological proof as a result of bronchoscopic biopsy, resection of the affected lung or post-mortem examination.
(*b*) There is radiological evidence of a tumour in the lung and histological proof of secondary deposits in lymphatic glands.

(c) A tumour, which in the opinion of the person performing the bronchoscopy is clearly carcinomatous, is seen through the bronchoscope but no material is removed for histological section.
(d) A consultant member of the hospital staff has made a firm diagnosis of bronchial carcinoma on clinical and radiological evidence.

Some of the cases in the last group have been confirmed by their response to radiotherapy.

Of the 123 patients whose sputa were examined, 39 were subsequently shown to be suffering from bronchial carcinoma. The methods by which the diagnosis was reached were as follows :

| | |
|---|---|
| (a) Histological examination of material removed from the tumour . . . | 15 |
| (b) Histological examination of secondary deposits . . . . . . | 4 |
| (c) Bronchoscopy without histological examination . . . . . . | 8 |
| (d) Clinical opinion only . . . . . . . . . . . . . | 12 |
| Total . | 39 |

The final diagnosis of the 123 patients in the series—so far as it is known at the time of writing— is shown below :

| | |
|---|---|
| Total patients . . . . . . . . . . . . . . . | 123 |
| Patients finally diagnosed as not suffering from carcinoma . . . . . | 67 |
| Patients finally diagnosed as suffering from carcinoma . . . . . . | 39 |
| Total not yet diagnosed at the time of writing . . . . . . . | 8 |
| Untraced . . . . . . . . . . . . . . . . . | 9 |

The results of sputum examination for carcinoma cells in these patients are shown in Table I. All sputum examinations were carried out without prior knowledge of the patient's condition, as only by this means was it possible to test the value of the method.

TABLE 1.—*Results of Sputum Examination for Carcinoma Cells in* 123 *Patients.*

| | Total. | Proved positive. | Final diagnosis negative. | Not yet diagnosed. | Untraced. |
|---|---|---|---|---|---|
| *Category* 1. No cells suggestive of carcinoma seen in sputum . . . . . . | 76 | 6 | 59 | 3 | 8 |
| *Category* 2. Atypical epithelial cells seen upon which no certain opinion can be given . . . . . . . | 13 | 3 | 6 | 3 | 1 |
| *Category* 3. Cells seen which are highly suggestive of carcinoma . . . . . | 23 | 20 | 2 | 1 | 0 |
| *Category* 4. Cells seen which constitute clear evidence of carcinoma . . . | 11 | 10 | 0 | 1 | 0 |
| | 123 | 39 | 67 | 8 | 9 |

Of the 9 patients untraced, 8 were out-patients in whose sputum no cells suggestive of carcinoma were found, and who did not return to the hospital. One was an in-patient in whose sputum atypical cells were found upon which no definite opinion could be given. He left the hospital soon afterwards and the final diagnosis is not at present known to the writer.

Of the 8 cases recorded as not yet diagnosed, 3 had no cells suggestive of carcinoma in their sputum, 3 showed epithelial cells which were considered to be atypical but were not thought to constitute evidence of carcinoma, 1 showed cells that were highly suggestive of carcinoma and 1, cells that were held to constitute clear evidence of it. The 6 in the first two groups are all patients investigated recently who are almost certainly not suffering from carcinoma but in whom no final diagnosis has been reached. The 2 patients who were diagnosed as sputum positive are both elderly men in one of whom there is strongly suggestive radiological evidence of carcinoma with persistent collapse of the right lower lobe but no tumour demonstrable on bronchoscopy. The other has a shadow persisting in the left mid-zone but has acid-fast bacilli in his sputum. It is thought quite probable by those in clinical charge of him that he has carcinoma as well as tuberculosis. No bronchoscopy has been performed.*

Of the 123 patients, there are 106 in whom a final diagnosis has been reached. The results of sputum examination on these 106 will be considered in the analysis which follows.

For the purpose of analysis, it is considered that Categories 1 and 2 constitute a negative, and 3 and 4 a positive sputum diagnosis, and the terms " sputum negative " and " sputum positive " are used in this sense.

It will be seen from Table I that in two cases, cells that were highly suggestive of carcinoma were seen in the sputum of patients who were finally diagnosed as not having suffered from the condition. One of these patients has now come to post-mortem at which a diagnosis of haemorrhagic bronchitis was made : the other, who was admitted complaining of haemoptysis, is now well, with no evidence of any pulmonary lesion. The films of the sputa of both these patients have been reviewed and a single clump of cells in the former would still be held to be highly suggestive of carcinoma. This is shown in Fig. 41. In the case of the latter patient, there was a large amount of rather eosinophilic epithelial debris in the film, the appearance being one which would not now be thought by the writer to be suggestive of carcinoma unless some indication of the malignant nature of the cells were also present. Of the two errors described here, the first must be held to be due to a limitation of the method, whereas the second was caused by lack of experience.

Of the 106 patients under review, 39 were finally diagnosed as suffering from bronchial carcinoma, and 67 from other conditions. The analysis of the results of sputum examination on these patients is as follows :

| | |
|---|---|
| Total cases in the series | 106 |
| Cases finally diagnosed as positive | 39 |
| Cases finally diagnosed as negative | 67 |
| Total positive diagnoses made by sputum examination | 32 |
| Correct positive diagnoses | 30 |
| False positive diagnoses | 2 |
| Percentage of negative cases incorrectly diagnosed as positive | 3·0 |
| False positive diagnoses expressed as a percentage of the total series | 1·9 |
| Positive cases in which carcinoma cells were not found in the sputum | 9 |
| Percentage of positive cases in which carcinoma cells were not found | 23·1 |
| Percentage of positive cases correctly diagnosed by sputum examination | 76·9 |

* *Addendum*—At the time of going to press, these two patients are still under observation. The one whose sputum contained cells that were considered clear evidence of carcinoma has now been readmitted to hospital a year after the cells were first found with clear clinical evidence of carcinoma with multiple bone metatases. His sputum now contains cells similar to, but more pleomorphic than those first found. The other, whose sputum contained cells that were thought highly suggestive of carcinoma has been treated for a year for pulmonary tuberculosis and has improved, though the radiological picture is little changed. The diagnosis of carcinoma, though unlikely, must still be considered.

*The Number of Specimens Examined.*

An analysis has been made of the number of specimens submitted from each patient. It would, of course, be ideal for a constant number to be sent to the laboratory in all cases but in practice this has been found difficult to achieve for several reasons, one of which is that bronchoscopy is often performed soon after admission, rendering specimens taken during the following week unsuitable for examination. Often, therefore, only two or sometimes one specimen is obtained. In addition, it has usually been found that only one sample is submitted from an outpatient or, if more are available, only one arrives in a suitable state for examination. Patients who have little sputum may only produce a single specimen in the course of many days or if more are available some are likely to consist wholly of saliva.

In Table II an attempt is made to relate the accuracy of diagnosis to the number of specimens examined per patient.

TABLE II.— *Number of Specimens Examined and Accuracy of Diagnosis.*

A. *Patients correctly diagnosed as sputum positive.*

| | | Percentage of total diagnosed. |
|---|---|---|
| Total patients | 30 | |
| Diagnosed as positive on first specimen | 20 | 66·6 |
| ,, ,, second ,, | 6 | 86·6 |
| ,, ,, third ,, | 1 | 90·0 |
| ,, ,, fourth ,, | 1 | 93·3 |
| ,, ,, fifth ,, | 2 | 100 |

Total number of specimens examined from these 30 patients = 49
Mean of number of specimens examined per patient before a positive diagnosis was made . . . . . . 1·63

B. *Patients correctly diagnosed as sputum negative.*

| | |
|---|---|
| Total patients | 65 |
| One specimen only examined | 36 |
| Two specimens examined | 17 |
| Three ,, ,, | 7 |
| Four ,, ,, | 2 |
| Five ,, ,, | 3 |

Total number of specimens examined from these 65 patients = 114
Mean of number of specimens examined in sputum negative patients . . . . . . . . . . 1·75

Thus, considering only the cases that were correctly diagnosed, 163 specimens were examined from 95 patients, giving a mean of the number of specimens examined per patient of 1·71. If two films are made from each specimen, and the time taken to examine each film is 20 minutes, then the time spent upon each patient is a little over 1 hour.

It has been stated earlier that it is often possible to indicate the predominant cell type in a bronchial tumour from sputum examination, and it is my practice to do this wherever possible. Unfortunately, histological confirmation of this finding is often lacking as in many cases no biopsy is performed. Among those in which there has been a histological examination, there has been agreement between histological and cytological diagnosis of the predominant cell type. Table III shows the results.

TABLE III.—*Correlation Between Cytological and Histological Diagnosis.*

| Diagnosis on sputum. | Total. | Histo-logically confirmed. | Histology does not confirm. | No histo-logical examination. | Not yet diagnosed. |
|---|---|---|---|---|---|
| Squamous cell | 16 | 7 | 0 | 9 | 0 |
| Oat cell | 6 | 3 | 0 | 3 | 0 |
| Adenocarcinoma | 1 | 0 | 0 | 0 | 1 |

Thus :

| | |
|---|---|
| Total cases | 30 |
| Predominant histological type stated in | 23 cases. |
| Histological confirmation was obtained in | 10 „ |
| No histological examination was made in | 12 „ |
| No final diagnosis has been reached in | 1 case. |

### CONCLUSIONS.

In the present series of 123 patients, there were 106 in whom the diagnosis was known at the time of writing. Of these, 39 were shown to be suffering from bronchial carcinoma and 67 had other conditions with no evidence of a bronchial tumour. Of those who had carcinomata, 30 (76·9 per cent) showed carcinoma cells in the sputum.

Sputum specimens from two patients in the series were wrongly reported as positive. One of these patients complained of haemoptysis but recovered rapidly and subsequently showed no evidence of any lung lesion. His sputum contained a great deal of eosinophilic cell debris which was thought to be the remains of necrotic carcinoma cells. The other patient suffered from severe haemoptysis and one sputum specimen showed the clump of cells apparently surrounding a vacuole which is shown in Fig. 41. He died and at post-mortem examination was diagnosed as having suffered from haemorrhagic bronchitis, no evidence of carcinoma being found.

The films from both patients have been reviewed. Those from the first would not now be considered suggestive of carcinoma as the cells showed no other appearance associated with malignancy. It is considered that this mistake was due to lack of experience. The clump of cells from the second patient would still be held to be suggestive of carcinoma if seen again, which indicates that the method has limitations. Further investigation is required into the significance of clumps of cells of this type.

It has been my experience that when a confident diagnosis of carcinoma can be made from sputum examination, it is usually possible to tell the predominant cell type in the tumour, and so far, when there has been an opportunity for histological confirmation, this has been shown to be correct.

It has been stressed that in a series of this type, it is possible that misdiagnoses of carcinoma may go undiscovered because the proportion of positive cases is high. It is not certain that there are no such mistakes in the present series but since only about one third of the patients were in fact suffering from bronchial carcinoma, the chances are in favour of such errors being discovered.

It is necessary that the limitation of a diagnostic technique of this type be clearly understood. A negative finding is of no significance in the exclusion of carcinoma, and may be due simply to the fact that the minute portion of the specimen chosen for examination contains no cells that can be recognised as malignant. This is likely to be the commonest cause of failure. Or the tumour may not be shedding any neoplastic cells into the lumen of the bronchus. It has already been pointed out that one false positive diagnosis has been made in the present series on the evidence of a clump of cells which resemble cells seen in carcinoma so closely that the writer finds it impossible to distinguish clearly between them. It would therefore be unrealistic to rely upon a method of this type in cases in which histological methods can be used.

The fact that sputum examination is not generally satisfactory during the first week after bronchoscopy has been performed makes it necessary that specimens be sent to the laboratory before this procedure is undertaken as otherwise, if no tumour is found at bronchoscopy, a week is wasted before specimens suitable for cytological examination can be obtained.

I believe the examination of the sputum for carcinoma cells to be of considerable value, and its use together with clinical evidence may provide the clinician with a means of diagnosing bronchial carcinomata which cannot be demonstrated by bronchoscopy. Its reliability is not yet absolute because of an occasional false positive diagnosis : it is clear that there is still an urgent need for further research.

### SUMMARY.

A review is given of some of the more important publications that deal with the demonstration of carcinoma cells in the sputum.

The method used in the preparation of sputum films in the Clinical Pathology Department at University College Hospital is described. All films are stained firstly with methylene blue, and those that are considered to contain cells that are suggestive of carcinoma, are also stained with haemalum and eosin. The relative merits of wet films and permanent preparations are discussed and a simplified method of making permanent films is described.

Descriptions and illustrations are given of the cells normally found in the sputum and of cells associated with carcinoma. Emphasis is laid upon the types of cell that may lead to a false positive diagnosis.

The results of the examination of sputum specimens from 123 patients are analysed. Of these, a final diagnosis had been reached at the time of writing in 106, 39 being shown to suffer from bronchial carcinoma. Cells which were either highly suggestive of carcinoma or were considered to be clear evidence of it were seen in the sputum of 30 (76·9 per cent) of these. An attempt is made to analyse the effect of the number of specimens examined upon the accuracy of diagnosis.

Where possible, it has been the writer's practice to state the predominant cell type when a positive report is made. This was done in 23 of the 30 cases so reported. An opportunity for histological confirmation has occurred in 10 of these, and there has been agreement.

Two sputa were erroneously thought to contain cells which were highly suggestive of carcinoma. The reasons for these two misdiagnoses are critically examined and it is concluded that one could have been avoided and one could not.

Further research is necessary to eliminate false positive diagnoses.

My thanks are due to Professor M. Maizels, Professor G. R. Cameron, F.R.S., and Dr. N. Schuster for their encouragement, help and criticism in the course of this work, to Dr. K. F. W. Hinson and Mr. A. W. Smart, senior technician in the Pathology Department of the London Chest Hospital, for my early instruction in the method, and to the clinical staff and sisters of University College Hospital, who made the material for this study available to me.

### REFERENCES.

ALTGAUZEN, A. Y.—(1939) *Klin. Med., Mosk.*, **17**, 90.
BAMFORTH, J.—(1946) *Thorax*, **1**, 118.
BARRETT, N. R.—(1938) *J. thorac. Surg.*, **8**, 169.
BETSCHARDT, E.—(1895) *Virchows Arch.*, **142**, 86.
DUDGEON, L. S.—(1936) *St. Thom. Hosp. Rep.*, **1**, 51.
*Idem* AND WRIGLEY, C. H. J.—(1935) *J. Laryng.*, **50**, 752.
FOOT, N. C.—(1952) *Amer. J. Path.*, **28**, 963.
FRIEDMANN, I.—(1951) *J. Laryng.*, **65**, 1.
GLOYNE, S. R.—(1937) *Tubercle*, **18**, 292.
GOWAR, F. J. S.—(1943) *Brit. J. Surg.*, **30**, 193.
HAMPELN, P.—(1887) *St. Petersburger Med. Wschr.*, **17**. (Cited by Wandall, H. H., 1944.)—(1897) *Z. klin. Med.*, **32**, 247.—(1918) *Mitt. Grenzgeb. Med. Chir.*, **31**, 672.
HERBUT, P. A., AND CLERF, L. H.—(1946) *J. Amer. med. Ass.*, **130**, 1006.
LIEBOW, A. A., LINDSKOG, G. E., AND BLOOMER, W. E.—(1948) *Cancer*, **1**, 223.
McKAY, D. G., WARE, P. F., ATWOOD, D. A., AND HARKEN, D. E.—(1948) *Ibid.*, **1**, 208.
PAPANICOLAOU, G. N.—(1942) *Science*, **95**, 438.—(1949) *Ann. int. Med.*, **31**, 661.
PERRIN, J., AND LITTLEJOHN, G. T.—(1950) *J. clin. Path.*, **3**, 40.
SCHUSTER, N. H.—(1947) IN DYKE, S. C.—" Recent Advances in Clinical Pathology." London (J. & A. Churchill Ltd.) p. 136.
SCHMIDTMANN, M., AND SAUER, R.—(1952) *Virchows Arch.*, **322**, 603.
WANDALL, H. H.—(1943) *Acta path. microbiol. scand.*, **20**, 485.—(1944) *Acta chir. scand.*, Suppl. 93, 1.

### KEY TO ILLUSTRATIONS.

For the purpose of description, the illustrations will be divided into the following sections :

A. Cells normally found in the sputum.

B. Cells which have proved to be reliable in the diagnosis of bronchial carcinoma :
- (*a*) Cells associated with carcinoma of predominantly squamous cell type.
- (*b*) Anaplastic carcinoma cells :
  - (1) Oat cells.
  - (2) Spheroidal and spindle cells.
- (*c*) Cells associated with tumours diagnosed histologically as alveolar cell carcinoma.

*Note.*—No illustration is at present available of cells from an adenocarcinoma in which there has been histological confirmation.

C. Cells which are considered to be inconclusive in the diagnosis of bronchial carcinoma.

D. Cells seen in non-malignant conditions which may lead to a false diagnosis of carcinoma.
All photographs are taken at a magnification of 400 diameters.

### A. *Cells Normally Found in the Sputum* (Fig. 1 to 7).

*Fig.* 1.—A group of three squamous epithelial cells. They have pale eosinophilic cytoplasm and a lightly staining nucleus which is more or less centrally placed. Where the cytoplasm is folded, the cell tends to glisten, a feature that is not always seen and appears to be associated with a greater degree of keratinisation in the more mature cell.

When the sputum is much infected, many bacteria may be seen in the cytoplasm of these cells, and on occasion, the cell body may be infiltrated with pus cells, giving an appearance which once led the writer to suspect carcinoma. See Fig. 39.

A normal squamous cell is well shown in the picture of oat cells, Fig. 27.

*Fig.* 2.—Squamous cells of a smaller type which tend to occur in sheets in the sputum. They stain more deeply than the larger type of squame, and are regular in size and rather angular in shape. There is very little variation in nuclear size, shape or staining density, a feature which usually makes them easy to distinguish from carcinoma cells.

*Fig.* 3.—Normal bronchial epithelial cells obtained by aspiration at bronchoscopy. Characteristically these cells are elongated with a ciliated free border and a filiform point of attachment to the basement membrane. The nucleus, which is regular in size and oval in shape, is nearer to the attached end than to the free border of the cell and tends to make a small expansion in the cell body. The chromatin pattern is fine in the freshly shed cell but becomes dense in an older specimen.

Sometimes, a mass of tangled bronchial epithelial cells gives a very disorderly appearance which may lead to confusion with carcinoma cells, but it is almost always possible to find some cells in the mass which possess cilia, the presence of which is a clear indication that the cells are benign. Rarely, multinucleate bronchial epithelial cells are seen.

*Fig.* 4.—Bronchial epithelial cells which show a considerable degree of irregularity in shape and size and dense misshapen nuclei. They either do not possess cilia or have lost them. Such an appearance is not infrequently seen in the sputum, and may lead to confusion with carcinoma. On close inspection, however, it will be seen that a number of cells in the clump have the general shape of bronchial epithelial cells, showing a wide free border and a filiform point of attachment, and the nuclear distortion is mainly due to the presence of vacuoles in the cytoplasm. It is possible to discern gradations from those which appear nearly normal to those which seem grossly abnormal. The whole clump can therefore be dismissed as benign.

*Fig.* 5.—Macrophages devoid of dust particles. A group of macrophages which show the general characteristics of these cells. The cytoplasm is usually strongly eosinophilic and often has a somewhat foamy appearance. The nucleus occupies less than half the cell area and may be round, flattened at one side or reniform. It is usually eccentric in position and is commonly right at the side of the cell. When it is reniform, the convex border is towards the periphery.

Normally the nucleus stains lightly and possesses a clearly defined perinuclear membrane and a small nucleolus. Multinucleate macrophages are common, occasionally as many as ten nuclei being found.

While the majority of macrophages are more or less circular, they do on occasion show a great variety of shapes and may become considerably elongated, bearing a superficial resemblance to a distorted epithelial cell.

*Fig.* 6.—Macrophages containing dust particles. This is the form in which macrophages are normally seen in the sputum. The presence of dust enables them to be identified immediately, provided that steps are taken to determine that the dust is actually in the cell and not overlying it. Carcinoma cells do not contain dust particles.

*Fig.* 7.—Lymphocytes and neutrophils.

Neutrophils in the sputum appear as they do in other situations. Usually they are mature, though occasionally immature forms may be seen.

Lymphocytes, when they are present, are commonly single, but may show clumping as in the illustration, in which case they bear a superficial resemblance to oat cells. The precise difference between the two will be noted when the latter cells are described. See Fig. 27 and 28.

The nucleus of the lymphocyte is constantly circular or very nearly so, and it occupies half or a little over half of the diameter of the cell. The nuclear chromatin is fairly dense and tends to be concentrated at the periphery. It has an uneven appearance, but no chromatin network is seen. Where cells appear in a clump, the nuclei maintain their round shape, not being distorted by those of neighbouring cells. The cytoplasm shows as a clearly visible moderately eosinophilic rim round the nucleus.

### B. *Cells which have Proved to be Reliable in the Diagnosis of Bronchial Carcinoma* (Fig. 8 to 31).

(*a*) *Cells associated with carcinoma of predominantly squamous cell type* (Fig. 8 to 26).

*Fig.* 8 *and* 9.—Films showing a number of carcinoma cells which are recognisable under the low power of the microscope because of the considerable degree of pleomorphism which they exhibit.

*Fig.* 8.—A field showing a clump of poorly differentiated squamous carcinoma cells. The large nuclei with uneven edges, the coarse chromatin pattern, and the irregularity in nuclear size and cell shape all indicate that these cells have been shed from a carcinoma.

*Fig.* 9.—A field containing a considerable amount of eosinophilic cell debris with a few intact cells, one of which is of bizarre appearance. The presence of eosinophilic debris of this type is highly suggestive of carcinoma, though of course it could occur in other conditions leading to extensive epithelial breakdown in the bronchial tree or upper respiratory tract.

The large cell has three nuclei and gives the impression of having inclusions in the cytoplasm. These features, combined with the unusual shape of the cell, constitute clear evidence of carcinoma. The strongly eosinophilic cytoplasm is also suggestive of carcinoma, but may occasionally be seen in other degenerate epithelial cells.

It should be noted that the appearance suggestive of inclusions seen in this cell differs from that shown in Fig. 21, 32, 33 and 36, in all of which the inclusion is clearly cellular. In the present case, the appearance suggests either that pieces of the cytoplasm of other cells have become enclosed, or the phenomenon is not in fact due to inclusion at all, but is caused by local differences in the refractive properties of different parts of the cytoplasm. The effect, when it is seen, stands out clearly in both the methylene blue and haemalum and eosin films, and the writer has never seen it in non-malignant cells. It is possibly associated with keratinisation, as films which show " bright cells " frequently show this effect as well.

*Fig.* 10 *to* 21.—Single cells of types which have proved reliable in the diagnosis of bronchial carcinoma.

*Fig.* 10, 11 *and* 12.—Cells showing distinctive nuclear characteristics.

*Fig.* 10.—A cell showing marked nuclear hyperchromasia. A single cell from a film that contained many of similar type. The most striking feature of the cell is the fact that the nucleus stains nearly black. Such a change may be seen sometimes in markedly degenerate non-malignant epithelial cells in the sputum, but taken in conjunction with the irregularity of the nucleus and its relatively large size, it has proved a reliable indication of carcinoma.

*Fig.* 11.—A large cell with pale staining cytoplasm and an elongated irregular nucleus. The nucleus is not so dense as in the previous specimen, but its very unusual shape is easily made out under the low power of the microscope. This type of cell and that shown in the previous illustration frequently occur together.

*Fig.* 12.—A giant mononuclear cell with a very large nucleus. Mere nuclear size is, in the author's experience, unreliable as an indication of carcinoma (see Fig. 37), but the very large size of both this cell and its nucleus and the coarse chromatin pattern make cells of the type shown reliable as an indication of carcinoma.

*Fig.* 13, 14, 15 *and* 16.—Cells which are distinctive by virtue of their unusual shape.

*Fig.* 13.—A " tadpole " cell. An elongated cell with a long filamentous tail. The nucleus shows no unusual characteristics and is situated in the wider " body " of the cell. Sometimes, chromatin masses are seen in the tail, and on occasion, two cell bodies, each containing a nucleus, are united by a long filamentous bridge. A drawing of one of these is shown in Fig. 26.

*Fig.* 14.—A cell similar to that shown in the previous illustration in which the nucleus appears to be in mitosis.

*Fig.* 15.—Two cells of unusual shape, with markedly eosinophilic cytoplasm, one of them resembling a " tadpole " cell in a field containing some eosinophilic debris, probably another carcinoma cell, and many macrophages.

*Fig.* 16.—An elongated cell with ill-defined nuclear detail, but with, apparently, further chromatin masses at each end. This is a common type of carcinoma cell.

*Fig.* 17 *and* 18.—" Bright cells ".

Cells of this type are not very commonly seen, but when they are present they stand out extremely clearly and can be recognised with ease. The writer has seen them only in cases of squamous cell carcinoma, and nothing resembling them has been seen in any sputum specimen from a non-malignant condition.

They appear considerably more refractile than do normal cells, and by virtue of this property, look bright when compared with the cells that surround them. When put slightly out of focus they shine brilliantly.

The high refractility of these cells is possibly associated with keratinisation, and may be related to the appearance shown in Fig. 1, in which normal squamous cells are seen to shine where the cytoplasm is folded.

This phenomenon, though best seen in the haemalum and eosin preparation, is seen in the methylene blue film as well.

*Fig.* 17.—A typical " bright cell " sharply in focus. The cell is more brilliant than those in its neighbourhood, the cytoplasm appears rugged, and the cell itself is surrounded by a greenish coloured band which is probably due to some optical property of the cytoplasm.

*Fig.* 18.—A " bright cell " slightly out of focus. Its brilliance is enhanced compared with the cells in the surrounding field. The coloured band which surrounds the cell stands out clearly. In addition to the cell described, there is a considerable amount of eosinophilic debris shown, similar to that seen in Fig. 9, which is taken from another part of the same film.

*Fig.* 19 *and* 20.—Cells showing a double edge.

The possession of a clearly defined double edge is a property occasionally seen in squamous carcinoma cells. It is not easy to know why this change should occur.

*Fig.* 19.—A large cell with the appearance of a double edge. There is some suggestion of vacuolation between the two " edges ". The nuclear relationships are difficult to make out, but the cell appears to have a number of nucleoli.

*Fig.* 20.—A smaller cell of similar type to that shown in the previous illustration among a collection of cells consisting chiefly of macrophages.

*Fig.* 21.—A cell showing other cells included within its cytoplasm.

This phenomenon is frequently seen in squamous carcinoma cells, but it should not be considered to be diagnostic of carcinoma unless the cells show some of the other attributes of malignancy, as phagocytosis is a property of macrophages, and a macrophage enclosing another cell may simulate a carcinoma cell closely (see Fig. 36). Often, when this phenomenon is seen, it is impossible to decide whether the enclosing cell is a macrophage or a carcinoma cell (see Fig. 32 and 33).

In the present case, however, the giant size of the cell and the large hyperchromatic nuclei make it clear that both the including and the included cells are carcinoma cells.

*Fig.* 22 *and* 23.—Clumps of cells.

The presence in the sputum of clumps of apparently abnormal epithelial cells must always give rise to a suspicion of carcinoma, but it is essential that such clumps be not considered as diagnostic of malignancy unless the individual cells composing them have other malignant characteristics. Two clumps which are considered to have such characteristics are shown in this section. The reader should refer also to the clump shown in Fig. 34, which is from the sputum of a patient diagnosed clinically as suffering from bronchial carcinoma, but which is considered by the writer to constitute insufficient evidence to enable a diagnosis of carcinoma to be made. The present writer now shows considerable caution in the interpretation of the significance of such clumps as two mistakes have been made. Before this it was thought that clumps of epithelial cells of this type constituted strong evidence of carcinoma. However, the clumps shown in Fig. 41 and 42, which were both thought to be strongly suggestive of carcinoma, were from patients who had no other evidence of carcinoma at the time, and in one of whom there was later no post-mortem evidence. The other has been followed for nine months and has developed no sign of a tumour.

*Fig.* 22.—A clump of cells showing considerable variation in nuclear staining. One of them has three nuclei. The individual cells might be confused with macrophages, but the latter are smaller and are not seen in clumps of this type, nor do they show such variation in nuclear characteristics.

*Fig.* 23.—A clump of cells some of which bear a strong resemblance to " tadpole " cells.

*Fig.* 24, 25 *and* 26.—Drawings of features seen in squamous carcinoma cells in the methylene blue preparation.

(b) *Cells associated with anaplastic carcinoma* (Fig. 27 to 31).

*Fig.* 27 *and* 28.—(1) *Oat cells.*

Oat cells, when seen in the sputum, tend to occur in clumps, and are approximately the same size as a lymphocyte. They have the following characteristics which, taken together, distinguish them clearly from any other cell that is seen in the sputum:

(1) The nucleus is large in relation to the cell. The cytoplasm may be visible as a thin rim, or it may not be seen at all. Where neighbouring cells are closely applied to each other, as is often the case, cell borders are not seen, the appearance being that of a syncytium.

(2) The nuclei of all the cells in a clump are of approximately the same size, but they vary considerably in shape, many of them tending to be rectangular. The reason for this is that the nuclei of neighbouring cells indent each other. This is the most important single feature in the identification of oat cells.

(3) The nucleus usually stains deeply, but the chromatin pattern can nearly always be distinguished. Under the $\frac{1}{12}$-inch objective it is seen to be reticulate, with small masses of chromatin here and there. It resembles closely the appearance of a reticulocyte stained to show the reticulum.

None of these features is seen in lymphocytes, which are the only cells found in the sputum with which oat cells are likely to be confused.

*Fig.* 27.—A small clump of oat cells which shows well the relative uniformity of nuclear size, and the variation in nuclear shape. The moulding together of the nuclei of neighbouring cells is clearly seen.

*Fig.* 28.—Part of a large clump of oat cells. The nuclear moulding is not so well shown as in the previous illustration, but the variation in nuclear staining density can be seen. Although most of the nuclei stain deeply, few are so dense that the chromatin pattern could not be made out under the $\frac{1}{12}$-inch objective.

The elongated arrangement of the clump is a feature commonly seen in oat cell carcinoma, and forms useful contributory evidence of the condition. Macrophages may occasionally be seen in similar compact clumps, the individual cells being compressed against one another but their nuclear characteristics are very different from those of oat cells and confusion is not therefore likely to arise. Oat cells can be identified rather more easily in the haemalum and eosin than in the methylene blue film, but their essential characteristics are shown in each.

*Fig.* 29.—(2) *Spheroidal and spindle cell carcinoma.*

A field showing a group of cells similar to, but larger than, oat cells and a small bundle of spindle cells. The nuclei of the spheroidal cells are mostly large and nearly fill the cell, but in some of the cells, the nucleus has contracted down and appears degenerate.

Spindle cells occur in bundles, a small one of which is shown in the illustration.

(c) *Cells associated with tumours diagnosed histologically as alveolar cell carcinoma* (Fig. 30 and 31).

In the writer's experience no one cell type is characteristic of this variety of tumour.

*Fig.* 30.—A cell of bizarre shape, containing three masses of chromatin. The general appearance of this cell suggests squamous cell carcinoma.

*Fig.* 31.—A field from a sputum film which shows clumps of cells, one of which exhibits vacuolation. Clumps of cells of this type have been described in adenocarcinoma.

### C. *Appearances which are Considered to be Inconclusive in the Diagnosis of Bronchial Carcinoma* (Fig. 32 to 34).

*Fig.* 32 *and* 33.—Cells showing other cells enclosed within them.

These fields are from sputum films of patients suffering from bronchial carcinoma of predominantly squamous cell type. They probably are carcinoma cells, but are not considered to constitute sufficiently strong evidence upon which to make a diagnosis, as macrophages showing inclusions can look very similar to this. See Fig. 36.

*Fig.* 32.—A binucleate cell encircling another cell. The neutrophils are probably superimposed.

*Fig.* 33.—A cell showing two cells enclosed within it. The lumpy appearance of the chromatin of these three cells makes it probable, but not certain, that these are carcinoma cells.

*Fig.* 34.—A clump of cells, the individual members of which have no definite characteristics of malignancy though one of the cells seems to possess large nucleoli—an appearance which may be due to the superimposition of pus cells. At one time, clumps of this type were thought by the writer to be diagnostic of carcinoma, but a clump of rather similar appearance led to a false positive diagnosis in a patient who was found at post-mortem examination not to be suffering from carcinoma. See Fig. 41.

For clumps of cells that are considered to be diagnostic of carcinoma, see Fig. 22 and 23.

### D. *Cells seen in Non-malignant Conditions which may Lead to a False Diagnosis of Carcinoma* (Fig. 35 to 43).

*Fig.* 35.—A group of epithelial cells showing a considerable degree of pleomorphism and nuclear degeneration. This is the most confusing appearance that has been seen in the whole series. The cells resemble carcinoma cells closely, but in no case is the nucleus large. This field, and the three that follow, were from the sputum of a single patient, in whom a positive diagnosis was made as a result. No treatment was instituted, and he has now been followed for over a year without any evidence of carcinoma developing.

*Fig.* 36.—A macrophage showing another cell included within it. This should be compared with the cells shown in Fig. 21, 32 and 33.

*Fig.* 37.—A group of cells, two of which have large nuclei with a course chromatin network. They are probably not macrophages, as the nuclei are too large. It is more likely that they are epithelial cells in an actively growing state.

*Fig.* 38.—Two cells showing vacuolation of their cytoplasm. They resemble some types of carcinoma cell in the possession of vacuoles, but they have the proportions of a macrophage. Cells of this type are relatively common in the sputum.

Vacuolated cells are not significant in the diagnosis of carcinoma unless they occur in clumps and possess some of the other characteristics associated with malignancy.

*Fig.* 39.—A squamous epithelial cell heavily infiltrated with neutrophils in a grossly infected sputum. This appearance, in which the epithelial cell is reduced to a thin rim round the neutrophils, is seen to occur in both normal epithelial cells and carcinoma cells.

*Fig.* 40.—A body which resembles the centre of an epithelial pearl, from the sputum of a patient who has developed no evidence of carcinoma in the course of nearly a year since this finding was made. It was probably derived from the epithelium of the mouth or pharynx, but its precise origin is not clear.

*Fig.* 41 *and* 42.—Clumps of cells.

*Fig.* 41.—A clump of cells which appear to surround a vacuole from the sputum of a patient who, on the strength of this evidence, was thought by the writer to be suffering from bronchial carcinoma. The patient came to post-mortem and was diagnosed as having suffered from haemorrhagic bronchitis. See also Fig. 22, 23 and 34.

*Fig.* 42.—A small piece of tissue with ill-defined cell boundaries and large vesicular nuclei, from the sputum of a patient who in the ensuing nine months has developed no evidence of carcinoma, though, of course, such evidence may appear later.